# Chemical pollution imposes limitations to the ecological status of European surface waters

**DOI:** 10.1038/s41598-020-71537-2

**Published:** 2020-09-09

**Authors:** Leo Posthuma, Michiel C. Zijp, Dick De Zwart, Dik Van de Meent, Lidija Globevnik, Maja Koprivsek, Andreas Focks, Jos Van Gils, Sebastian Birk

**Affiliations:** 1 National Institute for Public Health and the Environment (Centre for Sustainability, Environment and Health, DMG), PO Box 1, 3720 BA Bilthoven, The Netherlands; 2 Department of Environmental Science, Radboud University Nijmegen, Heyendaalseweg, Nijmegen, The Netherlands; 3 DdZ-Ecotox, Odijk, The Netherlands; 4 Mermayde, Groet, the Netherlands; 5 Faculty of Civil and Geodetic Engineering, University of Ljubljana, Jamova 2, 1000 Ljubljana, Slovenia; 6 Wageningen University and Research, PO Box 16, 6700 AA Wageningen, The Netherlands; 7 Deltares, P.O. Box 177, 2600 MH Delft, The Netherlands; 8 Faculty of Biology, Aquatic Ecology, University of Duisburg-Essen, Universitätsstr. 5, 45141 Essen, Germany; 9 Center for Water and Environmental Research, University of Duisburg-Essen, Universitätsstr. 5, 45141 Essen, Germany

**Keywords:** Biodiversity, Freshwater ecology, Ecology, Ecology, Environmental sciences

## Abstract

Aquatic ecosystems are affected by man-made pressures, often causing combined impacts. The analysis of the impacts of chemical pollution is however commonly separate from that of other pressures and their impacts. This evolved from differences in the data available for applied ecology vis-à-vis applied ecotoxicology, which are field gradients and laboratory toxicity tests, respectively. With this study, we demonstrate that the current approach of chemical impact assessment, consisting of comparing measured concentrations to protective environmental quality standards for individual chemicals, is not optimal. In reply, and preparing for a method that would enable the comprehensive assessment and management of water quality pressures, we evaluate various quantitative chemical pollution pressure metrics for mixtures of chemicals in a case study with 24 priority substances of Europe-wide concern. We demonstrate why current methods are sub-optimal for water quality management prioritization and that chemical pollution currently imposes limitations to the ecological status of European surface waters. We discuss why management efforts may currently fail to restore a good ecological status, given that to date only 0.2% of the compounds in trade are considered in European water quality assessment and management.

## Introduction

Human activities are a driving force influencing human health and the environment^1–3^. A variety of pressures may affect surface water quality and quantity, with impacts on aquatic life, ecosystem services and human health^4–12^. Chemical pollution is an increasingly important pressure^13–15^. In response, various regulations have been defined to prevent, assess and manage water quality. There is an increasing awareness that all pressures and impacts need to be considered at the systems-level^16^. For example, chemical safety needs to look at the whole ‘chemical economy’^17–19^, the multiple pressures on aquatic systems are assessed and managed starting from river basins and hydrological connections as the organizing principle^20–23^, and pressure impacts on biodiversity are evaluated in the context of large-scale land use patterns^24^. There is, however, no comprehensive system-level diagnosis of pressures and impacts. The assessment of chemical pollution pressures and impacts are globally separate from other pressures (e.g.,^25^). For surface waters, the separate assessment of chemical pressures can be recognized in, e.g., the EU-Water Framework Directive (WFD)^23^ and the U.S. Clean Water Rule^26^. The root cause for this phenomenon is that applied ecotoxicology could not copy the long-standing practices of applied ecology for the assessments of chemical pressures: analyses of field pressure-impact relationships are neither feasible (lack of such gradients in nature) nor ethical (to establish) for the multitude of compounds in trade (there are > 144,000 chemicals in trade in Europe, as registered by ECHA^27^). The separate assessment of the pressure and impacts of chemical pollution may result in poor diagnosis of causes of impacts on aquatic life and potentially unsuccessful management^28^. Indeed, the European Commission (EC) recently called for critical evaluations and improvements of the existing approaches to water quality assessment, to avoid ‘ill-founded measures and costly but ineffective management’^29^ and concluded that ‘… the key area where there is room to improve and to achieve better results is on chemicals’^30^. The problem of the current methods for separate assessment of chemical pollution and other pressures on surface waters may be bigger than currently perceived^15^, if it is considered that the current focus is (i) on few and not all chemicals (approx. 350 when considering European surface waters^31^, ≈ 0.2% of those in trade), (ii) on separate compounds and not mixtures^23^, and (iii) on current and not future emissions^13^. Scholars and the EC alike especially advocate considering the ‘universe of chemicals’^32^ and their mixtures^33^.

In this context, we aimed to evaluate (1) current practices in water quality assessment and management as well as (2) contemporary options to address chemical pollution of complex mixtures. We also looked ahead, at the potential of such options to be used in the comprehensive diagnosis of water quality that includes mixtures (not in the present paper). From a practical viewpoint, we base our work on available monitoring data used in water quality protection, assessment and management. We developed a case study on European surface waters and the Water Framework Directive (WFD^23^) as practical and regulatory context. The WFD environmental goals are to protect and restore good water body status. The latter is defined by the concepts of good ecological status and good chemical status, which are aggregated metrics that are derived from monitoring data (Supplementary Information—Section 1).

Here, for the first time, we characterize the chemical pollution pressure caused by complex mixtures on a European scale and we show that it is a factor that limits maintaining or reaching high or good ecological status. This was done based on a case study that expands on the Europe-wide pressure-impact study of Grizzetti et al.^4^, by adding chemical pollution information for substances of Europe-wide concern (so-called priority substances, PS)^34,35^. Although Grizzetti et al. paid attention to chemical pollution via a runoff proxy, they characterized it as representing chemical pollution neither comprehensively nor precisely^36^. In a stepwise way, we evaluate the current WFD method using protective water quality standards (1), the same method expanded with a mixture assessment step (2) and with effect-related rather than protection-related ecotoxicity data (3), and with a method that was designed to provide a quantitative chemical pollution pressure and impact characterization (4). Although the case study was developed with European data in the context of the WFD, the methods developed and tested in this study can be applied globally, as the proposed methods bridge the current gap between applied ecology and ecotoxicology in general.

## Results

### (1) Evaluation of Europe’s surface water quality with protective environmental quality standards

**Figure 1:**
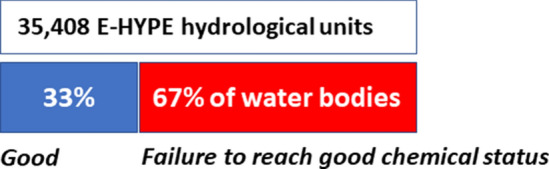
Evaluation of Europe’s surface water quality based on predicted environmental concentrations for 24 priority substances (PS) and the current water framework directive method for chemical status classification. Failure to reach good chemical status is assigned if the concentration of one or more PS in a hydrological unit exceeds its environmental quality standard. Outcomes based on the means of a year’s predicted environmental concentrations (mean-PEC), related to the approach used to characterize chemical pollution in the WFD, for 35,406 E-HYPE hydrological units.

Chemical pollution in the case study was assessed for 24 priority substances (PS), which are defined as compounds of EU-wide concern^23,37^ (Supplementary Information—Section 2). Based on predicted environmental concentrations (PECs) of these compounds for the whole of Europe (considering > 35,000 hydrological units, Supplementary Information—Section 2, and yearly mean PECs, Supplementary Information—Sections 3 and 4), we estimated that 67% of the water bodies would fail to reach good chemical status as defined in the WFD from current emission levels (Supplementary Information—Section 1; Fig. 1). In these hydrological units, the yearly mean concentration of one or more PS is predicted to be above its chemical-specific regulatory annual average environmental quality standard (EQS, Supplementary Information—Section 2). This is interpreted as a potential threat to aquatic ecosystems via direct exposures and/or exposures via secondary poisoning and/or a threat to human health (but note that the mere exceedance of an EQS does not directly inform the assessor which of the three endpoint(s) could be affected). The regulatory interpretation of this finding is that chemical emission reduction plans (primarily triggered at the EU-level) should result in maintaining protection for approx. 10,000 water bodies (blue), and counter—if possible—the potential threat(s) for the others (red). These outcomes align well with the recent analysis of status and pressures of Europe’s surface waters, where 62% of surface water bodies was classified as failing to reach good chemical status (considering measured concentrations of 45 priority substances^38^). Ours and the latter assessments differ by number of PS (24 versus 45) and the way to quantify exposure (predicted versus measured concentrations). The official WFD-pressures report, as well as water quality measurements, suggest explicitly that the results may under-estimate chemical pollution threats (see: https://www.norman-network.net/empodat/). It is not difficult to foresee that a high fraction—or even all—water bodies of Europe would fail to fall in the good status class if we would expand the judgment with a realistic number of chemicals emitted to the water system. Such a judgment can be made for > 13,000 compounds, given proposed protective quality standards for those^39^. Provided that the assessor would try to consider all the chemicals known to be locally emitted, the current WFD-indicator system shows whether the water quality objective (for a water body) is reached or not (blue or red) and (over an assemblage of water bodies) for which fraction of water bodies this holds or not (fraction of blue and red). The indicator is, however, not a quantitative metric that provides insights in impact magnitudes (as is the case for the ecological status, Supplementary Information—Section 1).

### (2) Similar as (1) but including a mixture assessment step

**Figure 2:**
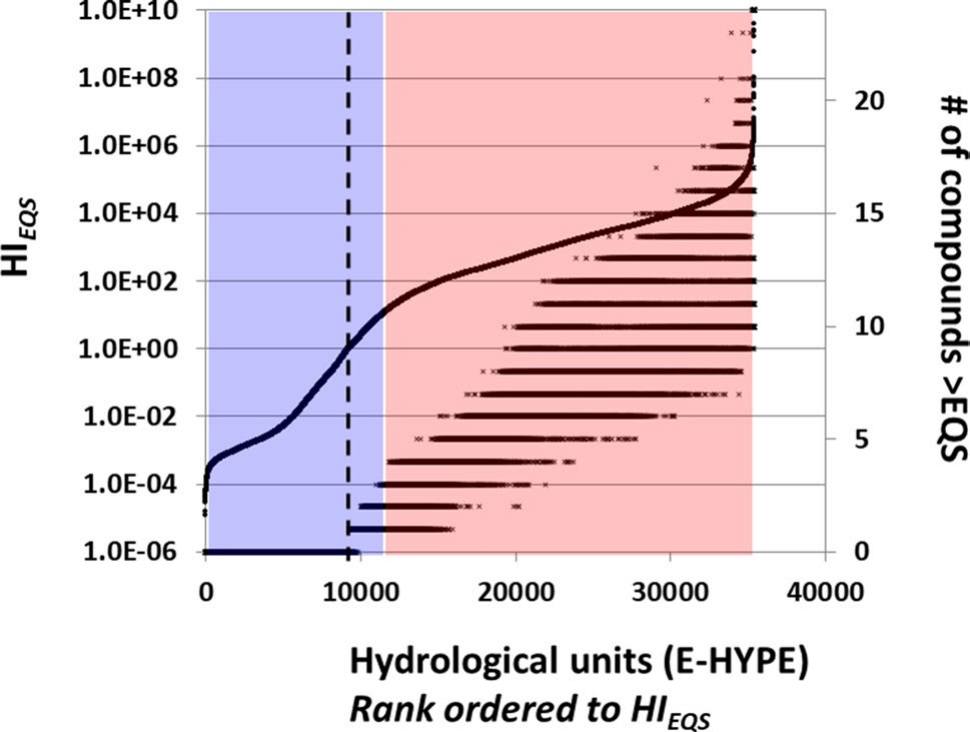
Evaluation of Europe’s surface water quality based on predicted environmental concentrations for 24 priority substances (PS) using a quantitative mixture pollution pressure metric, HI_EQS_. X-axis: E-HYPE hydrological units separately rank-ordered based on hazard indices (HI_EQS_, 1st Y-axis) and # of compounds (2nd Y-axis). 1st Y-axis: HI_EQS_. 2nd Y-axis: number of compounds contributing to the HI with an RQ > 1, plotted following the rank order of the pollution pressure described by HI_EQS_ (X-axis). Water bodies with similar HI_EQS_ or adjacent ranks can have different numbers of compounds that exceed their EQS. The transparent blue and red boxes are identical as in Fig. 1. Water bodies to the right of the dotted line are interpreted to be insufficiently protected against mixtures. *RQ *Risk Quotient, *HI* Hazard Index, *EQS* Ecological Quality Standard. Outcomes based on the means of a year’s predicted environmental concentrations (mean-PEC), related to the approach used to characterize chemical pollution in the WFD, for 35,406 hydrological units, as Fig. 1.

The optimization required for cost-effective management would commonly be based on a quantitative rank-order analysis of, for instance, the magnitude of damage, similar to Grizzetti et al.’s approach to rank-order the pressures they studied^4^. The results of our first analysis are, however, a water quality classification in two classes, which does not inform on the rank-ordered magnitudes of damage across water bodies. For that, applied ecotoxicology often uses the quotient of an exposure and an effect concentration (or effect threshold) for each compound, commonly called Risk Quotients (RQ)^40^. Mixtures are accounted for by summing the quotients over the compounds in the mixture, yielding a hazard index (HI = ΣRQ) to represent expected damage magnitude^40^. Using again the yearly mean predicted environmental concentrations and the EQSs as protective thresholds, we calculated all RQ_*EQS*_s and HI_*EQS*_-values for the hydrological units, and derived their cumulative distribution (Fig. 2, 1st Y-axis). We also derived the number of compounds contributing to such HIs (counting the number of compounds with an individual RQ_*EQS*_ > 1, 2nd Y-axis). HI_EQS_-values for mixtures > 1 indicate a risk level similar in interpretation as the ‘RQ > 1’-criterion for single chemicals in human health and environmental mixture assessments^40,41^. Note that the HI_*EQS*_ increases to a value of 1 for sites for which the EQS is not exceeded for any of the compounds (2nd Y-axis), suggesting insufficient protection for such mixture exposures. Given the projection of the transparent blue and red boxes from Fig. 1 on top of the HI_*EQS*_-distribution, the mixture assessment is indeed more sensitive than the current WFD assessment. Note also that the number of compounds causing failure to reach good chemical status can vary widely amongst water bodies (from none to all 24 compounds exceeding the EQS).

The HI_*EQS*_-values across hydrological units span more than fourteen orders of magnitude, and often the increased HI_*EQS*_ can be attributed to the RQ’s of 5 to 15 compounds, which would focus management attention on a large fraction of the studied PS to reach the status of sufficient protection. There is no sharp increase of mixture toxicity (HI_*EQS*_) at the projected EQS-based chemical class boundary. Apparently, if HI_*EQS*_ would relate to impact magnitudes, the current WFD-chemical status classification (Fig. 1) hides a large variability in the magnitude of expected impacts from mixture exposures. However, the summation of RQ_*EQS*_ has no scientific impact interpretation, as the RQ’s of different compounds are based on different endpoints (human health, direct ecological impacts or secondary poisoning impacts), different application factors (see Supplementary Information—Section 2), and different shapes and slopes of the underlying concentration-effect curves. An increased HI_*EQS*_-value may suggest increased pressure but also relatively poor information on the compounds’ (eco)toxicity^42^. The application factors (AF) of the studied chemicals used in deriving their EQS’s range from AF = 2 to AF = 50 (Supplementary Information—Section 2, Table S2). Thus, uncertainty on the (eco)toxicity of compounds co-determines part of the observed range of HI_*EQS*_*.* The regulatory valid interpretation of Fig. 2 is that HI_*EQS*_-values < 1 suggest a sufficiently high degree of protection for all three protection endpoints for the studied compounds and their mixtures. In such cases, management attention would focus on maintaining the good status by taking protective measures when e.g. land use (and associated emissions of chemicals) will change. As the quantitative interpretation of HI_*EQS*_ > 1 may have no specific scientific meaning, it is insufficient for use in a comprehensive pressure and impact analysis.

### (3) Evaluation of Europe’s surface water quality with effect-based hazard indices (again including the mixture assessment step)

**Figure 3:**
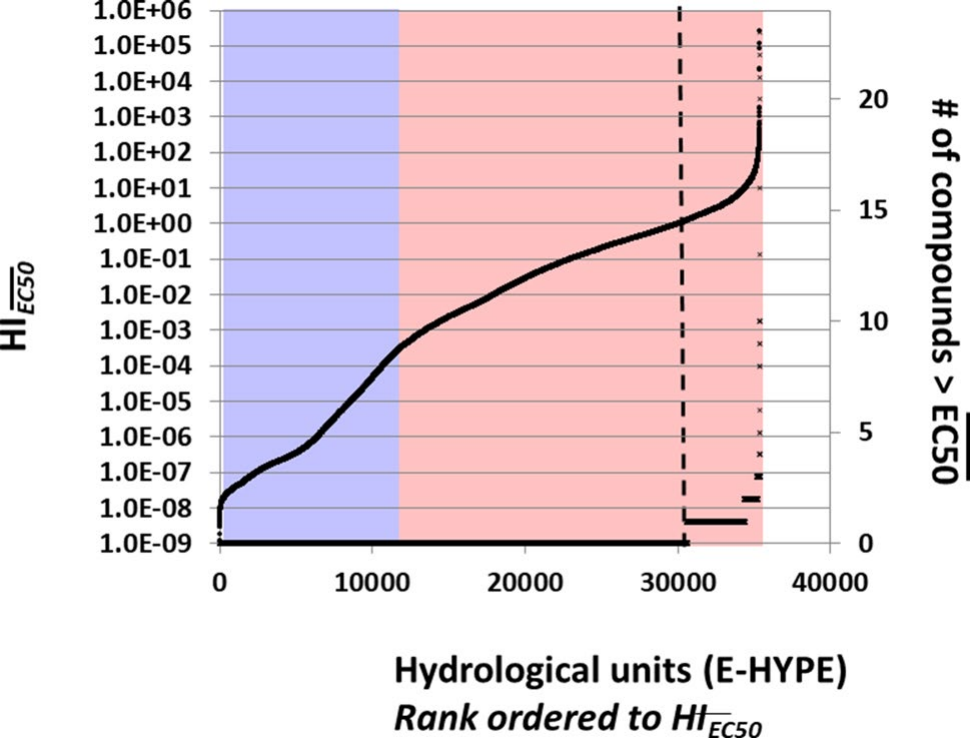
Evaluation of Europe’s surface water quality based on predicted environmental concentrations for 24 priority substances (PS) using a quantitative mixture pollution pressure metric, HI_EC50_. X-axis: E-HYPE hydrological units separately rank-ordered based on hazard indices (HI_EC50_, 1st Y-axis) and # of compounds (2nd Y-axis). 1st Y-axis: HI_EC50_. 2nd Y-axis: number of compounds contributing to the HI with an RQ > 1, following the rank order of the pollution pressure described by HI_Median-EC50_ (X-axis). The transparent blue and red boxes are identical as in Fig. 1. Water bodies to the right of the dotted line are interpreted to be exposed to a mixture causing an effect of 50% or more in a median-sensitive species. *RQ* Risk Quotient, *HI *Hazard Index, *EC50 *effect concentration causing an effect of 50%. Outcomes based on the means of a year’s predicted environmental concentrations (mean-PEC), related to the approach used to characterize chemical pollution in the WFD, for 35,406 hydrological units, as Fig. 1.

We solved the HI_*EQS*_-bias of the previous step (being: an EQS can relate to different impact endpoints, and can depend on different application factors; see Supplementary Information—Section 2) by employing the hazard index approach based on yearly mean concentrations and 50%-effect data (HI_*EC50*_). As each species in the aquatic species assemblage may have a species-specific sensitivity for a compound, we calculated the median EC50 (median-EC50, the mid-point of the fitted log-normal Species Sensitivity Distribution (SSD) curve^43^, the exposure concentration that would cause 50% effect on a vital endpoint such as growth and/or reproduction in half of the species) of a set of tested species for each compound to define the response metric used in the HI-calculation. The data analyses yielded the cumulative distribution of *HI*_*Median-EC50*_ for the studied hydrological units, with higher values related to higher predicted impacts on the ‘average-sensitive’ (but virtual) species, and likewise, higher impacts on field species. The outcomes suggest that the likelihood of substantial effects on species assemblages across European water bodies varies widely (Fig. 3, 1st Y-axis). The number of compounds that exceed the median-EC50 is (far) lower than those that exceed the EQS for a given watershed. Whereas nearly similar HI_*EQS*_-values could be attributed to few up till many compounds exceeding their EQS (resulting in the highly variable pattern in Fig. 2, 2nd Y-axis), the numbers of compounds exceeding the median EC50 are more consistent: an increase of HI_*Median-EC50*_ covaries with an increase in number of compounds contributing to it. At the dashed line (where HI_*Median-EC50*_ = 1), 15% of the water bodies would have a mixture exposure concentration that exceeds the 50%-effect level for the median-sensitive species. Substantial direct effects on growth and reproduction are to be expected on the median-sensitive species at that exposure level (and higher and lower impacts on more and less sensitive species in the field species assemblage). The overlay plotting of the blue and red boxes (as in Fig. 2) suggests the presence of small direct effects on species assemblages when the EQS of (at least) one compound is just exceeded (at the blue-red boundary). Note further that where HI_EQS_ = 1, the HI_Median-EC50_ is only about 1/10,000th of that.

Given that the WFD defines protection and—if needed—restoration as environmental goals, the results shown in Figs. 1 and 2 relate to the first goal, and Fig. 3 to the second goal. The latter figure is most closely related to the impact-related principle of the ecological status (Supplementary Information—Section 1). The HI_*Median-EC50*_-metric for chemical pollution appears to deliver relevant, quantitative information on the expected magnitude of possible impacts, so that it can be used as pollution pressure metric in a comprehensive multiple stress assessment in applied ecology^4,15,44^.

### (4) Does chemical pollution act as factor that limits maintaining or reaching good ecological status?

**Figure 4:**
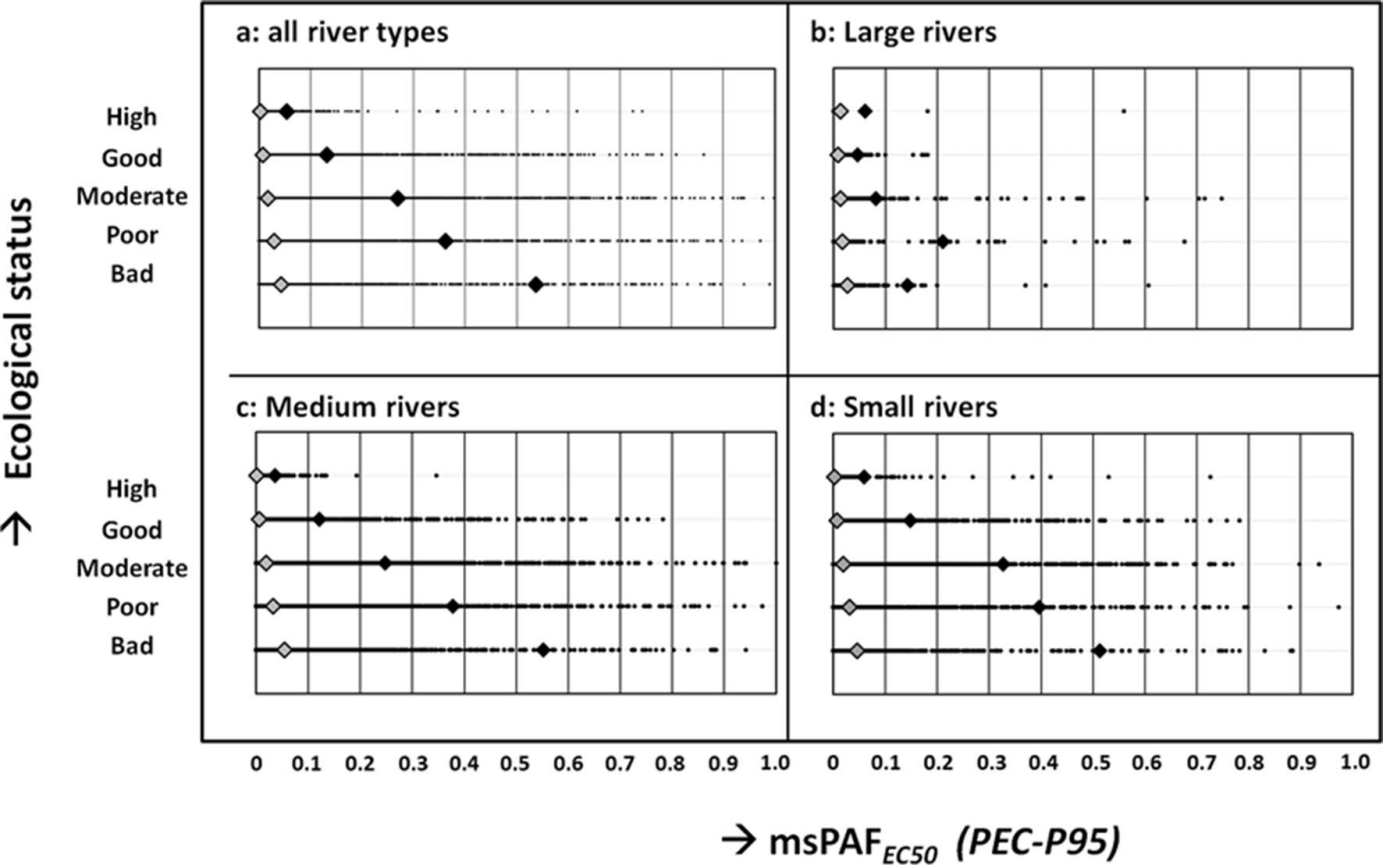
The relationship between chemical pollution pressure, expressed as predicted msPAF_EC50_ and ecological status for 46,977 European sites with matched chemical pollution and ecological status data. Panels show: results for all European sites (**A**), and for subsets of data for broad river types (RT) grouped as large (**B**), medium (**C**) and small (**D**) catchment size subsets. Data for river types RT8, 9 and 10 shown in Table 1. Catchment definitions: see Table 1. Dots: X = msPAF_EC50_, Y = ecological status class. Black diamonds: 95th percentile of msPAF_EC50_ value within the class. Grey diamonds: ibidem for 50th percentile. Outcomes based on the 95th percentile of a year’s predicted environmental concentrations (PEC_P95_).

We evaluated this question by using a chemical pollution metric that is conceptually preferable over HI_*Median-EC50*_, viz, the mixture toxic pressure. That is, the latter metric accounts for the phenomenon of non-linearity of the species sensitivity distributions (SSDs) used for this and it has the logical upper bound of 100% of species affected (whilst the HI-approach has no upper bound)^40^. This metric is expressed as the multi-substance Potentially Affected Fraction of species, here at the EC50-level (msPAF_*EC50*_)^45^. In short, under the general assumption that SSDs derived from available ecotoxicity data represent the sensitivity distributions of aquatic species, chemical-specific SSDs are used to quantify the per-compound PAF_ECx_ given an environmental concentration. Subsequently, those outcomes are aggregated with a mixed-model approach to the msPAF_ECx_, whereby values for compounds with similar and dissimilar modes of action are aggregated by assuming concentration and response additivity, respectively. As shown by citations to it in the literature, this metric has been used in hundreds of chemical pollution studies around the globe. As an example, if a mixture is calculated to cause a mixture toxic pressure (msPAF_*EC50*_) of 0.1, this implies that 10% of the tested species would be affected at or above the 50%-effect level if reared in water polluted with that mixture. This metric varies with economic activities and geographic characteristics (Supplementary Information—Section 7) and was not associated to other pressure variables potentially affecting European surface waters (Supplementary Information—Section 8). We evaluated whether chemical pollution acts as limiting factor for ecological status, analyzing raw data patterns and results of various statistical analyses. Because of the presence of pesticides in the list of studied compounds, we focused mainly on the 95th percentile of the predicted exposure concentrations (PEC_*P95*_, implying that exposures may be higher for approx. 18 days in a year), but evaluated also whether and in how far the choice of exposure metric matters for the conclusions derived in the present study (mean concentrations, as in Figs. 1, 2 and 3, or PEC_*P95*_, as in Fig. 4; see Supplementary Information—Section 9).

Figure 4 shows that the raw data distribution of msPAF_*EC50*_-values based on PEC_*P95*_-values ranged from near 0 to nearly 1 (X-axis, panel A: all sites), whereas the Y-values are the categorical ecological status classes (Supplementary Information—Section 1). Panel A shows the data for all spatially aligned (chemical–ecological) chemical pollution and ecological status data across Europe (46,997 XY-points). The other panels show the results for subsets of sites, discriminated via the classification of broad river types, by catchment size^46^. Table 1 summarizes the outcomes of the statistical analyses for separate broad river types (RT1–RT10, and for all sites together).

**Table 1:** Summary overview of relationship between chemical pollution pressure (msPAF_EC50_) and ecological status classes both for separate river types and for all data (lower right corner).

Lowland	Very large rivers		Lowland (> 100)		Lowland (< 100)			
Ecological status	RT1	#	RT3	#	RT2	#		
High	0.06	36	0.05	437	0.08	210		
Good	0.05	541	0.15	3,425	0.19	1,793		
Moderate	0.08	1,306	0.30	6,724	0.41	2,521		
Poor	0.21	525	0.44	2,518	0.45	1,019		
Bad	0.14	215	0.57	932	0.54	438		
Total (matched sites)		2,623		14,036		5,981		

Mid_Altitude	Siliceous (> 100)		Siliceous (< 100)		Calcareous (> 100)		Calcareous (< 100)	
Ecological status	RT5	#	RT4	#	RT7	#	RT6	#
High	0.01	416	0.03	389	0.09	87	0.10	201
Good	0.05	1,664	0.09	1,447	0.10	846	0.15	895
Moderate	0.09	1,855	0.16	1,092	0.22	1,414	0.19	850
Poor	0.22	884	0.24	443	0.24	465	0.27	197
Bad	0.48	161	0.44	94	0.32	154	0.32	49
Total (matched sites)		4,980		3,465		2,966		2,192

Highland and Mediterranean	Highland and glacier		Medit. perennial		Medit. temp./small		All rivers	
Ecological status	RT8	#	RT9	#	RT10	#	RT1-10	#
High	0.02	430	0.05	181	0.13	236	0.05	2,623
Good	0.05	1,910	0.09	1,396	0.36	1,392	0.13	15,309
Moderate	0.06	1,081	0.27	1,502	0.48	1,103	0.27	19,448
Poor	0.08	188	0.31	647	0.54	266	0.36	7,152
Bad	0.05	88	0.74	236	0.64	78	0.54	2,445
Total (matched sites)		3,697		3,962		3,075		46,977

*RT1* very large rivers, *RT2* lowland brooks and streams with catchment size smaller than 100 km^2^, *RT3* lowland streams and rivers with catchment size 100–10,000 km^2^, *RT4* siliceous mid-altitude brooks and streams with catchment size smaller than 100 km^2^, *RT5* siliceous mid-altitude streams and rivers with catchment size 100–10,000 km^2^, *RT6* calcareous mid-altitude brooks and streams with catchment size smaller than 100 km^2^, *RT7* calcareous mid-altitude streams and rivers with catchment size 100–10,000 km^2^, *RT8* highland and glacial rivers, *RT9 *Mediterranean perennial, *RT10* Mediterranean temporal or very small brooks.

The first column per river type is the 95th percentile of the msPAF_EC50_ within the ecological status class (as in Fig. 4, black diamond markers), and the second column is the number of sites per class. Outcomes based on the 95th percentile of a year’s predicted environmental concentrations for 46,977 sites.

Three observations can be derived from the results. First, each ecological status class harbors sites with highly variable mixture toxic pressures. A site with low chemical pollution (X) needs not have high or good ecological status. It may fall in the poor ecological status class due to limitations caused by e.g. nutrient pressures^4^. Second, but clearly visible, the upper right corner of each panel is relatively data poor. This indicates that chemical pollution acts as a factor that limits maintenance or reaching of good ecological status, following the interpretation common for quantile regression^47^: no high ecological status under high mixture toxicity pressure. Third, the latter interpretation is robust, as the pattern holds for different subsets of the data (different broad river types, separated as in Table 1, or in river-size related subgroups as in Fig. 4, and for different choices of the exposure metric, Supplementary Information—Section 9). The findings are statistically significant for all data subsets (Kruskal–Wallis tests, all P < 0.001). This is illustrated in Fig. 4 as grey and black diamonds, which represent the P50 and P95 of the msPAF-values within each of the classes, respectively). If it is assumed that the ecological status classes are not truly discrete (as the reported classes by the member states) but in fact a representation of a quantitative degree of impacts (as motivated in Supplementary Information—Section 1), our findings imply that an increasing toxic pressure implies a gradually increased likelihood of pollution impacts on aquatic ecosystems through direct exposure of the species.

As we found no collinearity between chemical pollution and other pressures (Supplementary Information—Section 8), our analyses (visual inspection of the raw data plots and the statistical tests) clearly point to mixture exposures acting as a significant limitation to maintain or reach high or good ecological status. The msPAF-metric can be used as pollution pressure metric in a comprehensive multiple-stress assessment in applied ecology, as it is quantitative in kind, and as it relates to impact magnitudes (this study)^4,15^.

## Discussion

Chemical pollution acts as a limiting factor for the ecological status of surface water bodies and—if expressed by a quantitative metric that accounts for mixture exposures—it can be evaluated in a comprehensive diagnostic analysis of surface water quality problems together with other pressures (results 3 and 4). Current methods do not allow to draw such conclusions (see results 1 and 2). The methods (to obtain results-types 3 and 4) can be employed for thousands of compounds and their mixtures, provided that exposure and ecotoxicity data can be collected. Methods to comprehensively characterize the environmental ‘exposome’, by measured and/or modelled data, as well as to characterize species sensitivity data and -distributions are currently under development or promoted^48–51^. The results are statistical association rather than causal evidence, but similar results were found for completely different geographies, mixtures and impact metrics^28^.

Our results should not be interpreted as a reason to discard the methods utilizing protective standards (EQSs in Europe). Taking a systems-perspective, there are four subsequent ways to protect human health and the environment from chemical pollution threats, namely sustainable chemistry to design intrinsically safe chemicals (‘benign by design’)^52,53^, chemical safety assessment to allow only sufficiently safe products on the market^18^, product life cycle assessment to select relatively benign products^54^ and—eventually—water quality assessment and management to protect and restore environmental quality (this study). The wealth of ecotoxicity data, and models to analyze them, can serve all these purposes, including the key role of protective standards in deciding whether chemicals can be used safely, and their equivalents used in water quality classification (the EQS in Europe). We therefore propose to keep the strengths of using the protective environmental quality standards as key step in practical water quality protection, assessment and management. This closely relates to the judgment needed to reach the WFD-goal of environmental protection. However, we also propose to add a second assessment step, to improve the utility of assessment outcomes for chemical pollution of surface waters. This would result in a tiered system in which the first step characterizes *that* a water body may be affected by any chemical or mixture, or not (Fig. 1). When concentrations exceed this threshold, water management authorities are alerted. Moreover, trends in the fraction of water bodies where no standards are exceeded can be evaluated, to establish adverse trends of increasing emissions or the success of management actions^38^. The second step links to the impact magnitude assessment that is also part of the ecological status classification. This step quantifies the relative *magnitude* of likely impacts of chemical pollution of a water body, it helps ranking polluted sites, and it identifies pollution hotspots and the chemicals contributing most to that. This is key for focusing cost-effective management. The advantage of the tiered system is that it both encompasses the regulatory principle of consistent protection goals across regulatory contexts (step 1: similar protection principles for chemical safety assessment and environmental quality assessment) as well as of the impact-magnitude related assessment of chemical and other pressures in relation to the WFD (step 2). The second step further provides an opportunity to solve the problematic separation of applied ecology and applied ecotoxicology for water quality assessment and management^28^.

Our study was triggered by and helps solving practical problems encountered with the current globally-used indicator systems for chemical pollution, which often heavily lean on protection standards (such as the EQS) and per-chemical evaluations. We recognize two serious practical tradeoffs in the current regulatory approach. The first is, that regional water authorities appear to be reluctant to monitor potentially relevant chemicals. That is, authorities could be inclined to monitor all chemicals potentially emitted to the surface water in their area, based on a systematic DPSIR (Driver-Pressure-Status-Impact-Response) analysis of the land uses in their area as suggested by the WFD^55^. This could easily lead to 100% of the water bodies classified as ‘not good’ if all identified compounds would be monitored (‘red’ in Fig. 1). This effect applies for example to Sweden, with 100% of its water bodies classified as ‘not good’ for chemical pollution^38^, while the ecological status of Sweden’s water bodies is relatively good. The EQS-based classification system is clearly highly sensitive for deterioration. Second, at the same time, the classification system is clearly insensitive for management that is successful. That is, a ‘good’ status for chemical pollution is reached only when *all* chemicals are present at a concentration below their standard. True risk reduction can go unnoticed (‘red’ remains ‘red’ in Fig. 1) if the assessor does not inspect the raw monitoring data on exposure concentrations. When only the classified results are inspected, this acts as reason to stop management investments that can—in fact—be effective. An indicator system that is highly sensitive to deterioration and insensitive to restoration, and therefore requires extensive explanations to understand assessment outcomes, is not helpful to focus and optimize risk reduction management. Practitioners’ experiences have shown the need to improve the indicator system for chemical pollution pressure assessment.

Apart from the need for improvement from the practical experiences, we found six further reasons to implement the two-step assessment approach. First, the history of drafting the WFD shows that the regulation explicitly asks for quantitative indicators, that would allow for cost-effective environmental planning at the EU-scale^56,57^. Second, the proposed quantitative method for chemical mixtures was deemed required, as stated in the same history^55^, be it that methods to quantitatively assess mixture impacts lacked at that time. Third, past and current scholars suggest to improve the diagnostic step, expanding on the current classification, in analogy to practices in human medicine^55,58^. The observation of ‘disease’ (step 1) would be followed by a refined assessment to steer the focus and intensity of the prescribed curation (step 2). Fourth, the toxic pressure assessment embodies an approach for mixtures, as advocated by both scholars and policy makers^32,59^. Fifth, its use would address concerns raised by scholars on the implementation of the WFD, when they reflect on the holistic, water-system level principles vis-à-vis evolved reductionistic practices of the WFD and its implementation^60,61^. And sixth, as key notion, the use of the quantitative hazard index or toxic pressure assessments is explicitly suggested by the WFD: Annex II states that assessors should use multiple lines of evidence “…to carry out an assessment of the *likelihood* that surface waters bodies …. will fail to meet the environmental quality objectives ….”. It also suggests that modeling can explicitly be one of the methods used for that. The mixture toxic pressure metric does exactly what is suggested in Annex II^49,62^, whilst it can be employed to characterize sufficient protection (with msPAF_*NOEC*_, with NOEC = No Observed Effect Concentration) as well as impacts (with msPAF_*EC50*_)^49^.

The quantitative assessment of chemical pollution has been illustrated in the present study with the limitations of studying direct effects on mixtures on aquatic ecosystems for only 24 priority substances of EU-wide concern. Further studies could quantitatively assess mixture impacts that are caused by more compounds, and by impacts that occur due to secondary poisoning and on human health. Explorations of this kind were recently made for human health impacts, and found feasible^63^. Ecotoxicity data for such assessments are available for more than 12,000 compounds^49^. Using those would limit the frequency of ‘false negatives’ in the assessment of chemical pollution pressures (currently neglecting 99.8% of the compounds in trade, and their mixtures)^31^. The feasibility of this has been illustrated for WFD-goals of protection and restoration by plotting relative mixture toxic pressure maps for Europe related to the emissions of nearly 2,000 compounds (based on msPAF_*NOEC*_ and msPAF_*EC50*_, respectively)^34,35,49^.

Most importantly, the empirical association between chemical pollution pressure and ecological impacts (Fig. 4) has been found more frequently^28,64,65^, for different geographies, mixture compositions and impact endpoints. This generally implies that mixture toxic pressure maps provide information relevant for management prioritization, to ‘most-affected sites’, followed by an analysis of ‘most important chemicals within sites’. Similar to the identification of important chemicals (2nd Y-axis in Figs. 1 and 2) the mixture toxic pressure of a site can always be dis-aggregated to identify the key compounds causing harm. Indeed, such studies show that assessments made for separate taxa elucidate that mixture exposures likely affect the majority of taxa to different degrees^64,66^. Another study suggested that the per-taxon responses to chemical pollutants in the field are more sensitive than the response of the highly-aggregated ecological status metric (as in Fig. 4), and may occur below the protective regulatory standards^67^. These results imply that our findings shown in Fig. 4 do not yet represent the most sensitive approach in impact diagnosis.

The present study does not yet show outcomes of a comprehensive pressure assessment for Europe, which would require in-depth analyses of the combined types of data of Grizzetti et al. and the chemical pollution data of the present study. Preliminary outcomes of such data for European waterbodies suggest that approx. one third of the variability of ecological status in European surface waters can be attributed to chemical pollution pressures^68^.

Our study considered European surface water bodies in the context of EU regulations, but there are no conceptual limitations to use quantitative chemical pollution assessment methods (3 and 4) elsewhere. The method to characterize mixture impacts applies to the assessment and management of local water, sediment and soil, for quantifying and reducing the chemical footprint of chemicals used in a region^69,70^, and to find pro-active and innovative solutions as called for in the UN-Global Chemicals Outlook^14^.

## Methods

### Spatial extent and amending the chemical pollution pressure

We aimed to determine the statistical association between the chemical pollution pressure from mixtures of compounds of EU-wide concern (X) and ecological status (Y). The case study for that expanded on the goals and methods of Grizzetti et al.^4^. That earlier study addressed human-induced pressures on EU surface waters characterized via hydromorphological and hydrological metrics (with 4 and 3 parameters, respectively), integrated land use data (2 parameters) and pollution data (nitrogen, phosphorous and diffuse pollution from urban runoff) as well as the ecological status of aquatic ecosystems as defined in the EU Water Framework Directive^23^. The diffuse pollution proxy did not cover chemical emissions from households and from agricultural and industrial land uses, and was considered a provisional parameter to represent urban runoff^36^. In our study, we collected ecological status and pressures data, and expanded that dataset with chemical pollution pressure data at the European scale for 24 priority substances. These were selected because they are considered as a pressure of EU-wide concern, such that they are a primary focus of EU-level regulatory efforts to prevent and limit chemical pollution of EU surface waters^23,37^. Moreover, the use of the ecological status as impact metric implies the selection of PS as study compounds (Supplementary Information—Sections 1, 2).

### Chemical pollution pressure (various pressure parameters, X)

The study focused on 24 chemicals emitted due to various human activities (households, industry and agriculture; compound details are in Supplementary Information—Section 2).

The quantification of the chemical pollution pressure requires (1) exposure data representative for Europe, to be combined with (2) effect-related data and mixture assessment. We judged options for the exposure assessment, and evaluated four options for the effects’ assessment step.

#### Exposure

Applied ecotoxicology commonly applies measured or predicted exposure concentrations for chemical and environmental risk assessments. In line with the other pressure parameters of Grizzetti et al. we preferred measured environmental concentration (MEC) data for the selected chemicals, but those were found to be of too low quality and quantity to cover the studied number of water bodies (Supplementary Information—Section 3). We therefore used predicted environmental concentrations (PECs), as common in chemical safety assessment regulations. PECs were obtained by an integrated model approach, as applied in landscape-level chemical risk assessments (Supplementary Information—Section 4)^71^. The E-HYPE hydrological model was used as a basis, covering 35,406 hydrological units^72^. We derived daily PECs and used the year’s 95th percentile of these data (the year’s PEC_P95_) for deriving site-specific chemical pollution pressure metrics^35^. A comparison of PECs and MECs showed that the PECs were useful for the present study, because differences in PECs across sites were orders of magnitude larger than PEC-prediction uncertainties (Supplementary Information—Section 5)^35^.

#### Effect data and impact metrics

Using the PEC data, we derived four pollution pressure metrics, based on various options to select no-effect assessment endpoints (such as the EQS) or test effect endpoints (such as the EC50): (1) per compound risk quotients testing mean concentrations vis-à-vis the EQS (with RQ_*EQS,i*_ = *PEC*_*i*_/EQS_*i*_ for each compound *i*), (2) *ibidem* for mixtures, defined by HI_*EQS*_ = ΣRQ_*EQS*_ over the 24 compounds, (3) *ibidem*, but using the test endpoint EC50 rather than the no-effect related EQS (HI_*Median-EC50*_ = ΣRQ_*Median-EC50*_), and (4) the mixture toxic pressure with the latter test endpoint, expressed as msPAF_*EC50*_ (msPAF = multi-substance Potentially Affected Fraction) based on PEC_*P95*_ (to account for peak exposures by pesticides which are poorly represented in mean-PEC assessments) and compound-specific species sensitivity distributions (SSDs)^49^. Data for (1) and (2) were obtained from the regulatory dossiers (Supplementary Information—Section 2), and for (3) and (4) from collated EC50 data sets (following^45,49,73^). The HI- and msPAF-metrics were derived by applying established mixture models^40,45^. All mixture metrics were designed to represent the relative across-site differences in the potential of mixtures to cause harm, though they represent different regulatory or scientific principles. HI-outcomes for a set of water bodies range from zero to very high, going from clean to severely polluted sites, but are higher for HI_*EQS*_ than for HI_*Median-EC50*_, due to the focus on more sensitive endpoints and the role of the application factors (in analyses (1) and (2)). The mixture toxic pressure metric is expressed as multi-substance Potentially Affected Fraction (msPAF_*EC50*_) of species for a selected benchmark concentration and varies between 0 and 1 (none or 100% of species in a species assemblage predicted to be affected to the 50% effect level). In contrast to the assumptions for the HI-based mixture metrics, the msPAF-metric accounts for the non-linearity of the SSDs, whilst optionally accounting for different modes of action of chemicals in a mixture based on mixture assessment rules for that (concentration additivity applied within groups of compounds with similar modes of action, and response additivity across groups of compounds with different modes of action)^49^. In the present study, the assessments of assessment (1) to (4) were made by consistently assuming concentration addition as mixture model. The numerical differences in step (4) between the common approach and this approach are numerically negligible^19,74^.

### Ecological status data (response parameter, Y)

We collected data from the Water Information System for Europe (WISE) database for the reporting year 2010^75^. Data consist of the ecological status classification reported by EU-countries (not containing raw monitoring data). The spatial units are functional elementary catchments (FECs, average size approx. 60 km^2^). Based on FEC-outlets, we assigned ecological status and broad river type classes (Table 1) to entire FECs^76^. The ecological status classification is standardized across Europe and across the biological quality elements considered in the WFD (aquatic flora, invertebrate fauna, fish fauna)^77^. The ecological status is classified as high, good, moderate, poor or bad^23^. The classification implies that the Y-values used in the analysis steps of case study generally represent no deviation from water body type-specific reference conditions, and negligible, small, moderate or large impact on one or more biological quality elements. The use of the ecological status classes as impact metric comes with a source of potential bias due to the potential influence of non-priority substances (chemicals that are not listed as being of Europe-wide concern). This was judged to have a small effect on the final study outcomes, see Supplementary Information—Section 1 for further detail).

### Aligning chemical pressure- and ecological status data

The chemical pressure- and the ecological status data were aligned utilizing map coordinates of FEC and E-HYPE units (one E-HYPE unit may have various FECs). The thus combined data consists of 46,977 data lines, referred to as sites (Supplementary Information—Section 6). For each site the following data were available: site identity, broad river type, catchment area, altitude, the chemical data expressed as RQ_*EQS*_, HI_*EQS*_, HI_*Median-EC50*_ and msPAF_*EC50*_ (used as pressure variables, X) and ecological status class (used as response variable, Y). Upon alignment, both the pressure and the ecological status metrics represent highly aggregated information. The use of the year’s modelled mean-PEC or PEC_*P95*_ represents a time-integrated exposure assessment for a site, similar to the ecological status metric. The use of the PEC_*P95*_ for a compound means that exposure can be higher than this concentration for approx. 18 days of a year. Note that the simultaneous presence of each compound at their PEC_*P95*_-level at a site is unlikely to happen in reality, so that the X-parameter in the case study should be interpreted as a relative metric of the potential of the mixture at a site pose harm via chemicals. The aggregation of the time-variable exposures to one site-specific value (with different metric values representing increased likelihood of impacts) had, however, to be implemented because the site’s ecological status data are also singular, time-integrating metrics that result from all time-variable pressures on the biological quality elements. We operationally selected the year’s mean-PEC and PEC_*P95*_ to derive insights in the relative differences of mixture pressure across the sites.

### Data analyses

The data analyses consisted of various pre-assessments (statistics on parameter variation and covariation), followed by the stepwise assessment of the relationship between chemical pollution pressure metrics and impact metrics (methods 1–4). Results of some descriptive data analyses are in Supplementary Information—Section 7. The outcomes of a collinearity test (between chemical pollution pressure and other pressures) suggested that our final interpretation step is not or minimally biased by collinearity of known pressures (Supplementary Information—Section 8). In the final analysis step, we evaluated the mixture toxic pressure—ecological status relationship for the whole data set and for various subsets of data (discriminating via a broad river type classification). The latter was done to evaluate robustness of the outcomes, and to evaluate the presence of potential differences caused by different water-type specific ecosystem vulnerabilities^78^. We plotted and interpreted the raw XY-data for all sites and subsets of sites and tested for significant differences of the mixture toxic pressure distribution between ecological status classes via Kruskal–Wallis tests. We visualized whether and in how far increased mixture exposures implies a limitation to maintain high or good ecological status based on methods derived from quantile regression^47^. We evaluated, finally, whether the choice of the exposure metric (PEC_*P95*_) matters for the key conclusions (see also Supplementary information—Section 9). Based on our assessments, and tests made elsewhere, we demonstrated that other choices for characterizing exposure would imply only minor changes in the relative rank order of sites regarding the mixture pollution level^49^.

## Supplementary information

Supplementary Information.

## Data Availability

Data are available from the corresponding author on reasonable request.
