# Supplementary material for: Chemical pollution imposes limitations to the ecological status of European surface waters
Source: Sci Rep. 2020 Sep 9;10:14825. doi: 10.1038/s41598-020-71537-2 (PMC7481305; doi:10.1038/s41598-020-71537-2)
Supplement: Supplementary file 1 — Supplementary Information. [file 41598_2020_71537_MOESM1_ESM.doc]

**Chemical pollution imposes limitations to the ecological status of European surface waters**

Leo Posthuma*^1,2^

Michiel C. Zijp ^1^

Dick De Zwart ^3, 4^

Dik Van de Meent ^2, 4^

Lidija Globevnik ^5^

Maja Koprivsek ^5^

Andreas Focks ^6^

Jos Van Gils ^7^

Sebastian Birk ^8,9^

* Corresponding author, [leo.posthuma@rivm.nl](mailto:leo.posthuma@rivm.nl)

**Authors and Addresses:**

^1^ National Institute for Public Health and the Environment (Centre for Sustainability, Environment and Health, DMG), PO Box 1, 3720 BA Bilthoven, The Netherlands

^2^ Radboud University Nijmegen, Dpt. Environmental Science, Heyendaalseweg, Nijmegen, the Netherlands

^3^ ddz-ecotox, Odijk, the Netherlands

^4^ Mermayde, Groet, the Netherlands

^5^ University of Ljubljana, Faculty of Civil and Geodetic Engineering, Jamova 2, 1000 Ljubljana, Slovenia

^6^ Wageningen University & Research, PO Box 16, 6700 AA Wageningen, the Netherlands

^7^ Deltares, P.O. Box 177, 2600 MH Delft, The Netherlands

^8^ University of Duisburg-Essen, Faculty of Biology, Aquatic Ecology, Universitätsstr. 5, 45141 Essen, Germany

^9^ University of Duisburg-Essen, Center for Water and Environmental Research, Universitätsstr. 5, 45141 Essen, Germany

Supplementary Information (SI)

1. Selecting data for the present study
2. **Data types needed**

We selected data for the present study with an eye on the scientific goals and the utility of the outcomes of our study to water quality assessment and management practices under the European Union Water Framework Directive[^1^](#_ENREF_1), i.e., we aimed to start with monitoring data as must be collected and reported to implement the WFD.

Data types needed for the present study are metrics for chemical pollution pressure (X) and for aquatic ecosystem impacts (Y) at the EU-level. Data made available for practice at this level consist of highly aggregated data collected from monitoring a suite of water quality parameters of water bodies across Europe. Required data would preferably consist of raw monitoring data on pressures and impacts. However, Member States report data on pressures and impacts in highly aggregated formats[^2^](#_ENREF_2).

Relevant for the present study are the chemical status and the ecological status, as those are used in practice to characterize water quality and to trigger protective or restorative management action.

1. **Practical uses and availability**

The WFD is an action-oriented regulatory framework. That is, water quality management is triggered if a water body is not in a good quality status. A lower than good status implies an obligation for water quality management planning and to implement an adaptive management cycle up till the water quality is (at least) good. Key parameters used in this practical assessment-management cycle are the chemical status and the ecological status. Both are aggregated metrics. Solely these aggregated metrics are reported by Member States and were thus available for the present study. We aimed to use these as basis for our evaluations of current practices in pressure and impact assessment, and for exploring opportunities to improve on current practices (main text).

The chemical status is determined on the basis of monitoring data on those chemicals for which the frequency of occurrence and their expected risks are of Europe-wide concern. Those are referred to as priority substances (PS)[^3^](#_ENREF_3). Management attention is triggered primarily at the level of the whole union, given their EU-wide occurrence. Chemical status data are available in the format of the two classes: good chemical status, and failure to reach good chemical status. The latter class is assigned if the concentration of at least one PS exceeds its protective environmental quality standard (EQS).

Management action towards the PS is triggered at the EU-level for each compound that is listed as PS. The concentrations of the priority substances are monitored, and reported as chemical status classification, to evaluate whether measures taken result in increased numbers of water bodies in good chemical status. It is noted that an exceedance of the EQS may imply insufficient protection for three situations: direct impacts of exposure of aquatic life, impacts that are caused by secondary poisoning, and human health impacts. Due to these three exposure-impact pathways underlying the regulatory EQS and thus the chemical status classification, we could not use the chemical classification itself as pressure metric on aquatic ecosystems (X) for the present study. Therefore, we developed and evaluated the utility of four metrics to characterize the chemical pollution pressure of European water bodies (summarized in the next SI-Sections).

The ecological status is determined on the basis of monitoring data on biological quality elements (organism groups) and monitoring data on supporting hydromorphological and physicochemical quality elements. The ecological status of a water body can be classified as high, good, moderate, poor or low. The classes are defined as in Supplementary Table S1, whereby the ecological status is the lowest of either the assessment of the biological quality elements or the supporting quality elements (known as the ‘one out, all out’ principle). Management attention is warranted if any of the quality elements is affected, even when the ecological status is not yet diagnosed to be affected by a known pressure.

Supplementary Table S1. Summary of the key elements that define ecological status of a water body according to the normative definitions of WFD Annex V. BQE=Biological Quality Element.

| **Ecological status** | **Biological Quality Elements (BQE)** | **Supporting Quality Elements** | |
| --- | --- | --- | --- |
|  |  | **Hydromorphology** | **Physico-chemistry** (including basin-specific chemical pollutants, but *excluding* priority substances) |
| High | Undisturbed conditions; no, or only very minor, evidence of distortion | No, or only very minor, anthropogenic alterations | Values correspond totally or nearly totally to undisturbed conditions |
| Good | Slight deviation from undisturbed conditions; low levels of distortion resulting from human activity |  | Values ensure ecosystem functioning and allow for BQEs in good status |
| Moderate | Moderate deviation from undisturbed conditions; moderate signs of distortion from human activity | Conditions consistent with BQEs in good or worse status | Conditions consistent with BQEs in moderate or worse status |
| Poor | Evidence of major alterations; biological communities deviate substantially from undisturbed conditions |  |  |
| Bad | Evidence of severe alterations; biological communities normally associated with undisturbed conditions are absent |  |  |

1. **Advantages and disadvantage of the available data**

There are two scientific advantages of basing the study on the principles of the chemical and the ecological status classification: they represent independent pressure and impact parameters, and the impact parameter is a quantitative metric calibrated across biological organism groups and European regions (next to the fact that these classifications are used for water quality assessment and management practice).

The pressure and impact metrics, when based on priority substances and ecological status, are independent by their definition. The priority substances of EU-wide concern are *not* considered in the classification of the ecological status (Supplementary Table S1). Management of chemicals of Europe-wide concern is triggered solely by the chemical status class with a primary responsibility at the EU-level, and management of other pressures, including the non-priority substances, is solely triggered at the river-basin scale (or more refined scales) by the ecological status classification.

The ecological status classification represents a useful impact metric, as it is based on monitoring data which have been aggregated to an impact-related score for the biological quality elements (Supplementary Table S1, column 2) in an intensive inter-calibration process in the European Union. That is, after that process the hundreds of bioassessment systems that exist in Europe have been harmonized in order to provide a uniform definition of (no-)impact levels across European regions and organism groups. Other impact metrics (e.g., raw monitoring data on taxon abundances) are likely (far) far more diverse, e.g. if a taxon does not occur across Europe or is determined to different taxonomic levels. The nature of the impact metric (Y) as quantitative calibrated metric for impact magnitude is an advantage for the present study.

The indicator system of the WFD also has a disadvantage for the present study. That is, the moderate ecological status class can, in part, be populated by water bodies in which the concentration(s) of non-priority substance(s) exceed(s) the compound-specific EQS(s); such substances are referred to as ‘specific pollutants’ (commonly evaluated at the river basin scale, as river basin specific pollutants). Such water bodies are classified as being in moderate ecological status, without necessarily also showing an ecological impact (Supplementary Table S1). In these cases, the calibrated impact magnitude advantage (mentioned above) is ‘disturbed’ by a motive (related to triggering management) but that is itself not an observed impact on any biological quality element. We found that 8% of the studied water bodies that would be classified as being in high or good ecological status were classified as ‘moderate’ due to such ‘specific pollutants’. This outcome resembles the officially reported value for a similar data compilation (5% of the water bodies)[^6^](#_ENREF_6). The fraction of water bodies with a potential bias for the present study is thus low. Given the assessment results (see main text), the potential source of bias may be removed from the assessment, by removing all data points with the Y-value defined as ‘moderate’. Their removal does not change the conclusions, as can be judged by ‘virtual removal’ of the moderate class data in main text Figure 4.

1. **Data used**

Combination of the criteria of (1) utilizing available (aggregated) monitoring data, as those reported for the whole of the European Union and those related to assessment and management practices, (2) using ecological status data to represent an inter-calibrated metric to characterize impact magnitudes on aquatic life (at an aggregated level), and (3) focusing on priority substances to represent chemical pollution pressure (X, independent of the ecological status classification, X) resulted in the collection of ecological status data at the European scale, and on deriving various chemical pollution pressure metrics (X). Details on the latter are in SI-Sections 2, 3 and 4.

1. Studied chemicals: identity, characteristics, regulatory aspects

We selected 24 of the currently 45 priority substances (PS) for the present study, not being metals and not referring to priority substances that themselves are listed and evaluated as a subgroup of various substances.

The identities and the substance-related information used in the case study are listed in Supplementary Table S2.

- Column 1: Substances are operationally assigned to groups of similar kinds and likely similar modes of action[^7^](#_ENREF_7), which is information that can be utilized in the mixture toxic pressure modelling[^8^](#_ENREF_8).
- Column 2 to 4: Chemical name, Chemical Abstract System (CAS) number and molecular weight (MW).
- Column 5 to 13: Information on the regulatory protective environmental quality standard (EQS). We used the AA-EQS (Annual Average EQS):
  - Column 5: the value of the regulatory standard, the AA-EQS (in μg/L);
  - Column 6 to 11: Background information on the derivation of the AA-EQS; an AA-EQS is the lowest (most protective) threshold concentration derived from data for three protection goals, followed by the use of an application factor (AF) to lower the threshold concentration for chemicals with limited or low-quality data:
    - - (i) to protect aquatic ecosystems when chemical exposure directly affects species on test endpoints such as growth and reproduction,
      - (ii) *ibidem*, when chemical exposure affects species through secondary poisoning, and
      - (iii) to protect human health.
    - Column 6 to 7: Information on compounds for which the direct effects of the chemical on test species are the most sensitive protection endpoint. The ecotoxicity threshold may be derived via various data analysis methods (SSD=Species Sensitivity Distribution; EqPart=Equilibrium Partitioning; NOEC=No Observed Effect Concentration, MAC=Maximally Acceptable Concentration), combined with an AF;
    - Column 8 to 9: Information on compounds for which indirect effects via secondary poisoning are the most sensitive protection endpoint. The ecotoxicity threshold is derived by application of a bioconcentration factor (BCF) to effect study data, combined with an AF;
    - Column 10 to 11: Information on compounds for which the assessment of human health effects (H) is the most sensitive endpoint combined with an AF; for the studied chemicals, the threshold concentration relates specifically to carcinogenicity.
  - Column 12 to 13: Reference to a published regulatory EQS-derivation dossier (year of publication, and WFD/EQS report number, if available).
- Column 14 to 15: The parameters of the compounds’ species sensitivity distribution, derived from EC50-data on various tested species used for quantifying the stressor variable (X) in the present study[^7^](#_ENREF_7).
- Column 16: Across-species median EC50-value (midpoint of the log-normal SSD-EC50); this is the estimated EC50 of a ‘median-sensitive virtual aquatic species’, as derived from available ecotoxicity data from tested aquatic species[^7^](#_ENREF_7).
- Column 17: The ratio of the across-species median EC50-value and the regulatory AA-EQS.
- Column 18: Presence (Y) or absence (N) of measured environmental concentrations in the EMPODAT database of measured concentrations in European surface waters (<http://www.normandata.eu/empodat/>).

Supplementary Table S2. Substances and substance-related information sorted according to assigned subgroups of chemicals and assigned primary modes of action (MoA) names. CAS=Chemical Abstract System; AA-EQS=regulatory Annual Average Environmental Quality Standard (EQS); EcoDir = the EQS is derived from ecotoxicity data on direct effects on aquatic ecosystems as most sensitive endpoint; EcoInd = *ibidem* the EQS is derived from indirect effects on aquatic ecosystems (secondary poisoning); H = *ibidem*, the EQS is derived from toxicity data on human health effects as most sensitive endpoint; EQS-dossier = cited WFD-EQS dossier and its evaluation dossier # (all reports found via web searches); SSD-EC50-parameters are the mean (μ) and standard deviation (σ) of log_10_ transformed EC50 data; HC50-EC50 is the median EC50 as derived from a species sensitivity distribution of a compound (HC=Hazardous Concentration). MOA, μ, σ and HC50-EC50 were taken from literature[^7^](#_ENREF_7).


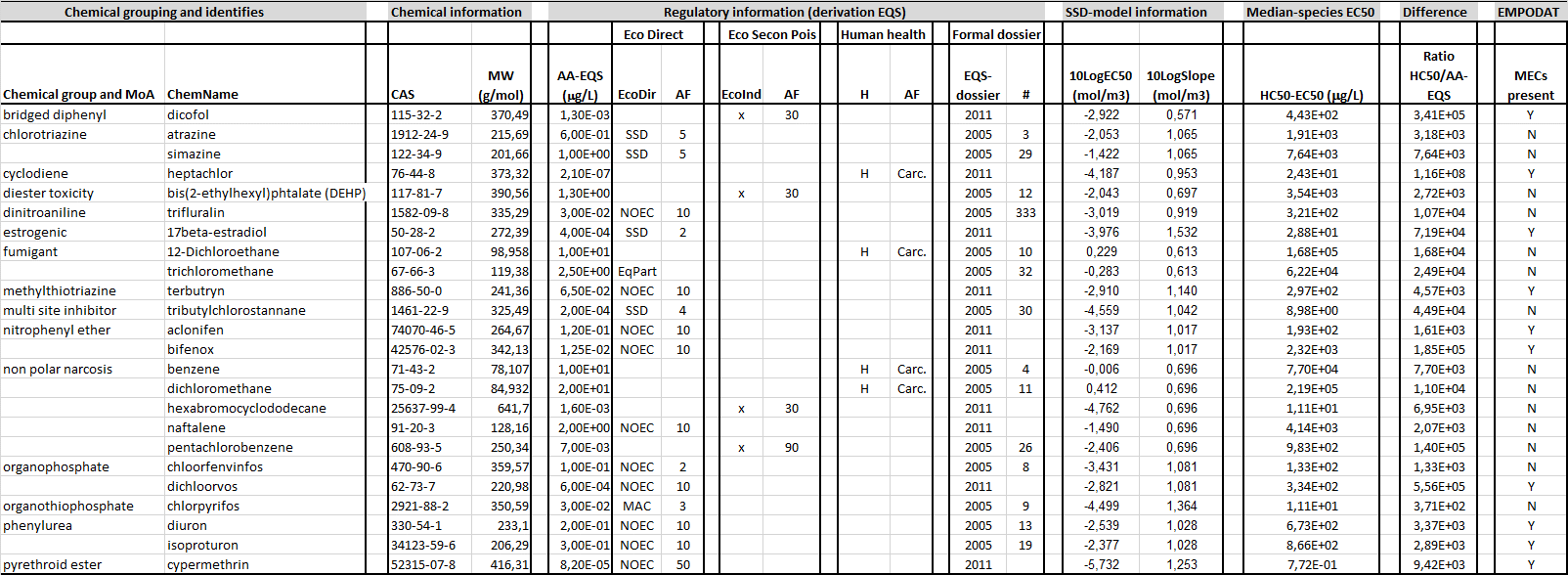


1. Exposure to (mixtures of) Priority Substances
2. **Options for concentration metrics for the present study**

Risk assessments for chemicals are commonly based on predicted and/or measured environmental concentrations (PECs and MECs, respectively): PECs for prospective chemical safety assessments (e.g., REACH[^10^](#_ENREF_10)), and MECs in the evaluation of water quality based on monitoring data (e.g., the WFD[^1^](#_ENREF_1)). A chemical is considered to pose potential harm when the PEC expected from foreseen use of the chemical exceeds the protective benchmark (PNEC = Predicted No Effect Concentration used in REACH[^10^](#_ENREF_10)). A water body is considered insufficiently protected when the MEC exceeds the EQS (the Environmental Quality Standard used in the WFD). The derivation of PNECs and EQSs is largely harmonized across chemical and environmental regulations to improve consistency amongst the different management frameworks, using the same level of protection. For the present study we aimed to collect exposure concentrations for the selected study compounds, and we evaluated in various steps whether there are enough MECs and/or PECs of sufficient quality and representativity for the European scale of the study.

1. **MEC data collection and evaluation**

Data collection. Measured environmental concentrations were obtained from a dedicated European database with monitoring data on emerging chemicals (NORMAN[^12^](#_ENREF_12), <http://www.normandata.eu/empodat/>, selection criteria: “chemical identity” and “river water”, download February 20, 2018). We added literature data from regional studies when available.

Results. Results are summarized in Supplementary Table S3. Data were found for only 10 of the 24 priority substances, yielding 409,483 MEC data points. For most of the measurements, the MEC was below the reported Limit of Quantification (LOQ). The compound with the highest fraction of data with a measurement >LOQ was diuron (24% of samples had a measured concentration >LOQ), and the compound with the lowest fraction was bifenox (0.2% of samples >LOQ). Compound data with a low frequency of MEC > LOQ and/or a narrow range of measured values near the LOQ are marked grey. The number of MECs for these compounds is insufficient for the present study. This holds to a lesser degree for four compounds, characterized by variation of MECs far higher than the LOQ, while also measured more frequently. Of these, only two compounds (diuron and isoproturon) are represented with approx. 15,000 MECs. The two other compounds (17beta-estradiol and terbutryn) have far less than 1,500 MECs, with relatively low variation of MECs for 17beta-estradiol. For compounds with relatively high numbers of MECs exceeding the LOQ, the measured range of concentrations spanned up to four orders of magnitude.

Utility of measured concentration data for the present study. As priority substances are defined as compounds of recognized EU-wide concern, we expected their MECs to be available in sufficient quality and representativity for the selected PS but found too few MEC-data for the case study purposes. We therefore collected and analyzed PEC-data, we used the MEC data to put the PECs into context, and we ran the final case study analyses with PEC-data (analyses below).

Supplementary Table S3. Summary of measured environmental concentrations (MECs) of studied compounds in European surface waters (source: EMPODAT). Compounds with less than 2% of the measurements exceeding their LOQ are marked in grey cells; they are characterized by lower measured exposure variability ranges than the other chemicals. LOQ = Level of quantification.


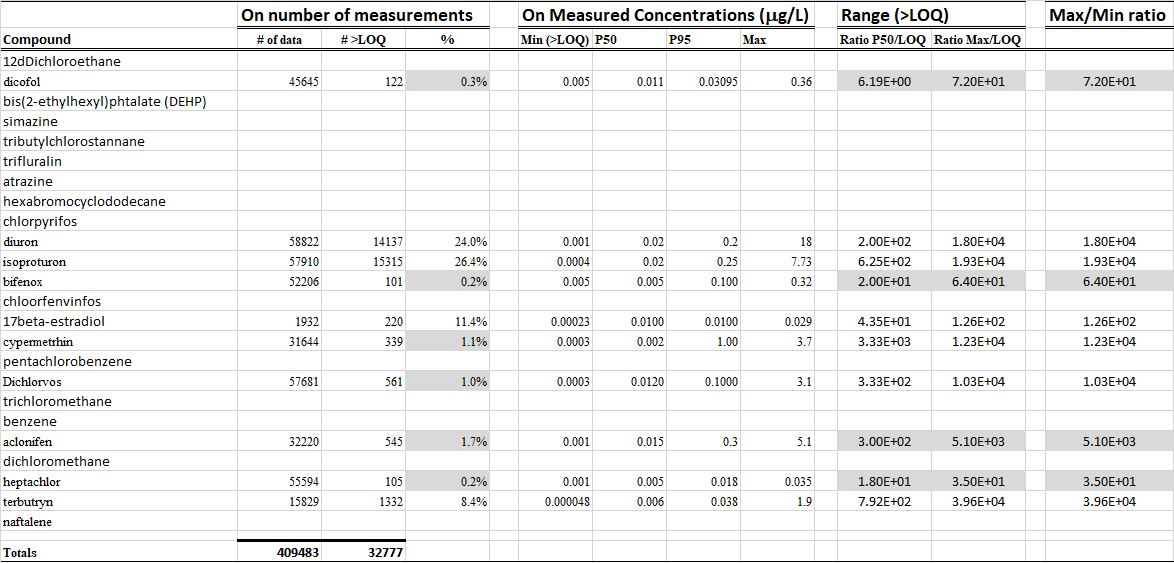


1. Predicted Environmental Concentrations

Approach. PECs were derived with a predictive model that was designed to increase our understanding of issues related to emerging chemicals in Europe’s river basins and to complement information and knowledge on MECs derived from field data[^13-15^](#_ENREF_13), similar to models developed for chemical pollution assessment and management elsewhere[^16^](#_ENREF_16). The model is based on the Europe-wide hydrology model E-HYPE[^17^](#_ENREF_17). The model was used to quantify 365 daily PECs for 35,406 hydrological units for each chemical (yielding ≈ 1.06*10^10^ PECs), using weather data from 2013.

Results. PEC distribution statistics are summarized in Supplementary Table S4, presenting the maximum, mean and minimum concentration of a compound for each hydrological unit within the modeled year (from 365 PECs per unit). The table provides information about PEC-differences amongst chemicals, over time (Min, Med, Max) and amongst hydrological units, which all differ by orders of magnitude. For example, the horizontal ranges of concentration percentile (P) values represent the concentration distribution across the hydrological units, whereby the P95/P5 range suggests inter-site PEC (exposure level) differences of up to fourteen orders of magnitude for a compound across Europe. Comparison of PECs to EMPODAT’s LOQ-levels (Supplementary Table S3) shows that few compounds would have detectable concentrations in a median-polluted water body (e.g., aclonifen), but that compounds would likely be detected in samples taken from less than 5% of the water bodies, but most often even less frequently.

Interpretation. The PEC data cover the same spatial range as the ecological status data (detailed below). The concentrations for most compounds in most hydrological units and for most sampling dates would not be quantifiable as measured environmental concentration (MEC) via surface water monitoring, due to limitations imposed by the LOQs.

Supplementary Table S4. Overview of Predicted Environmental Concentrations derived with a predictive model. Grey cells are PECs that are higher than the LOQ-level of the EMPODAT database, for the 10 compounds for which EMPODAT contains MECs (Supplementary Table S2).


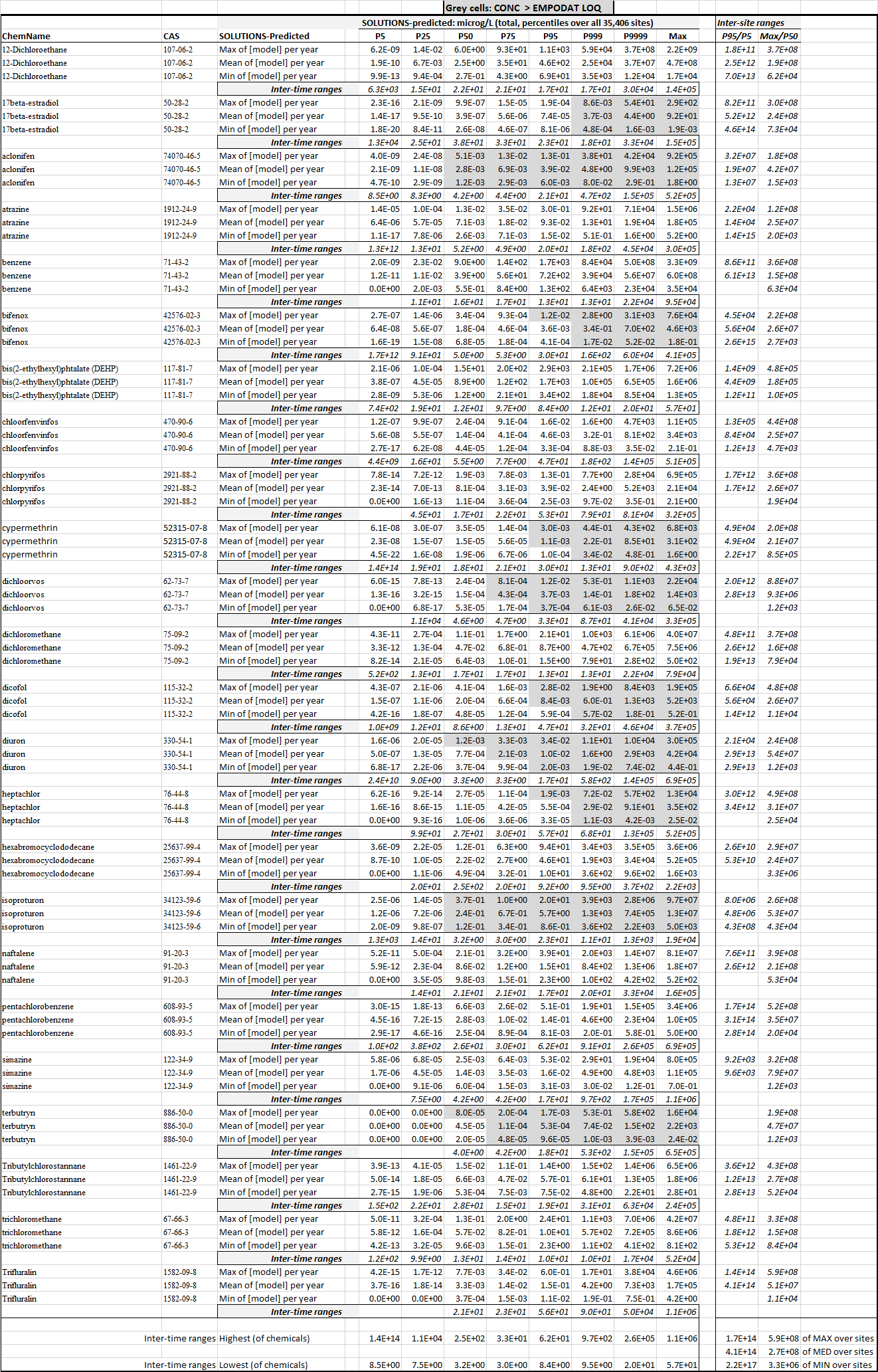


1. Comparisons, and motivation for selecting PECs
2. **Technical comparisons**

MECs and PECs can be seen as complementary information on chemical pollution[^13-15^](#_ENREF_13). We judged whether our PEC and MEC data represent useful information on chemical pollution of European surface waters, by comparing PEC and MEC data, and by evaluating PEC-patterns per se.

Approach 1, considering the 24 studied priority substances.

The PECs, covering all days of a year and all E-HYPE hydrological units, were compared to EMPODAT’s MECs. These comparisons considered whether and in how far PEC and MEC exposure level ranges show overlap.

Results approach 1. For all compounds with measured data, PECs overlapped with MECs (Supplementary Table S5). Overlapping concentrations were marked by a bold cell boundary in the table, when there was a maximum of one order of magnitude difference between PEC and MEC. We pragmatically chose the latter, given that exposures may vary over time due to varying economic activities and emissions, related to e.g. run off due to rain events, or due to the use of pesticides related to agricultural activities. The results suggest that the predicted exposure levels characterized by the PECs have all been observed, somewhere and at some sampling moment, in Europe. For some chemicals PECs and MECs are relatively similar for mean PEC-values (e.g., 1,2-dichloroethane, naphthalene, pentachlorobenzene), whereas for other chemicals higher-exposure sites relate best to MECs (e.g., DEHP).

Approach 2, literature data on the validation of the model tool.

Further information on PEC and MEC comparisons was obtained from a comparison made in other studies[^13-15^](#_ENREF_13). For these studies, high-quality monitoring data were collected for five European basins for as many chemicals as feasible (not necessarily being priority substances). Details are in the studies.

Results approach 2. The comparisons between co-located PECs and MECs – consisting of 246 compound/site matches of MECs and PECs – showed that the data on over- and under-predictions of PECs in comparison to MECs were equally distributed, and that over- and under-prediction remained within one order of magnitude for 65% of basin/substance combinations and within two orders of magnitude for 90% of basin/substance combinations. The range of under- and overprediction should be considered in the context of the trans-European PEC-ranges, which span many more orders of magnitude. Moreover, they should be considered from the viewpoint that both the MEC and the PEC are uncertain estimates of true (but unknown) exposure concentration variability in space and time (a MEC may differ by various orders of magnitude for a hydrological unit due to e.g. a pesticide spray event).

1. **Selection of PECs as basis to derive mixture metrics for the present study**

The results of both comparisons of PEC and MEC ranges showed that PECs overlap with but are not identical to the ranges of the MECs. Available MEC-data are, however, available in a far too fragmented form, with quantification problems for most compounds. In the evaluation of PECs for the present study, we found that PEC-values might occur in Europe (Approach 1) and that the PEC-model results are validated for selected compounds with under- and overpredictions much smaller than European exposure level ranges (Approach 2). Moreover, the prediction accuracy of the PEC-model resembles that of an independently developed model for pharmaceuticals[^18^](#_ENREF_18). This set of PEC-evaluation findings suggests that the target of the present study – the association between the level of stress possible imposed by chemical pollution and ecological status, despite PEC uncertainty – can be based on meaningful inter-site differences in PEC-based chemical pollution impact metrics.

A necessary step is creating a summary of the exposure over time. Because the response metric in the main study is the ecological status class determined for a year, resulting from pressure-impact relationships between pressures and impacts on biological quality elements, we also had to derive site-specific summary metrics for chemical pollution representing exposure during a year. In relation to the WFD-practices, we utilized mean PECs per water body (following the regulatory practices under the Water Framework Directive) and evaluated those vis-à-vis the Environmental Quality Standards (manuscript, Figure 1 and 2) and a calculated EC50-value for a median-sensitive species (the midpoint of a log-normal species sensitivity distribution, median-EC50, manuscript text Figure 3). Based on expert judgement, to account for pesticides showing peak exposures not well-represented by mean PECs, and in view of typical test conditions for various species groups lasting multiple days, we operationally selected the year’s P95 PECs to characterize exposure for the final evaluation (main text, Figure 4). This means that this exposure is present for at least 18 days (the 95^th^ percentile of 365 days). These PECs are used to characterize relative differences in chemical exposure levels across hydrological units in the further steps of the study. Note that any other value could have been selected, but that data evaluations suggested only limited effects of different choices for exposure characterization in inter-site exposure characterization differences[^7^](#_ENREF_7) (see also Supplementary information Section 9).

Supplementary Table S5. Comparing PECs and MECs, presented as log(MEC/PEC). Grey cells related to LOQ’s as in Supplementary Table S3. PEC and MEC values that differ within one order of magnitude difference are indicated by thick-bordered cells. The maximum-MECs were used to derive log(MEC/PEC), given frequent proximity of MECs to LOQs, Last column: E = EMPODAT MECs, L = Literature MECs[^19-21^](#_ENREF_19).


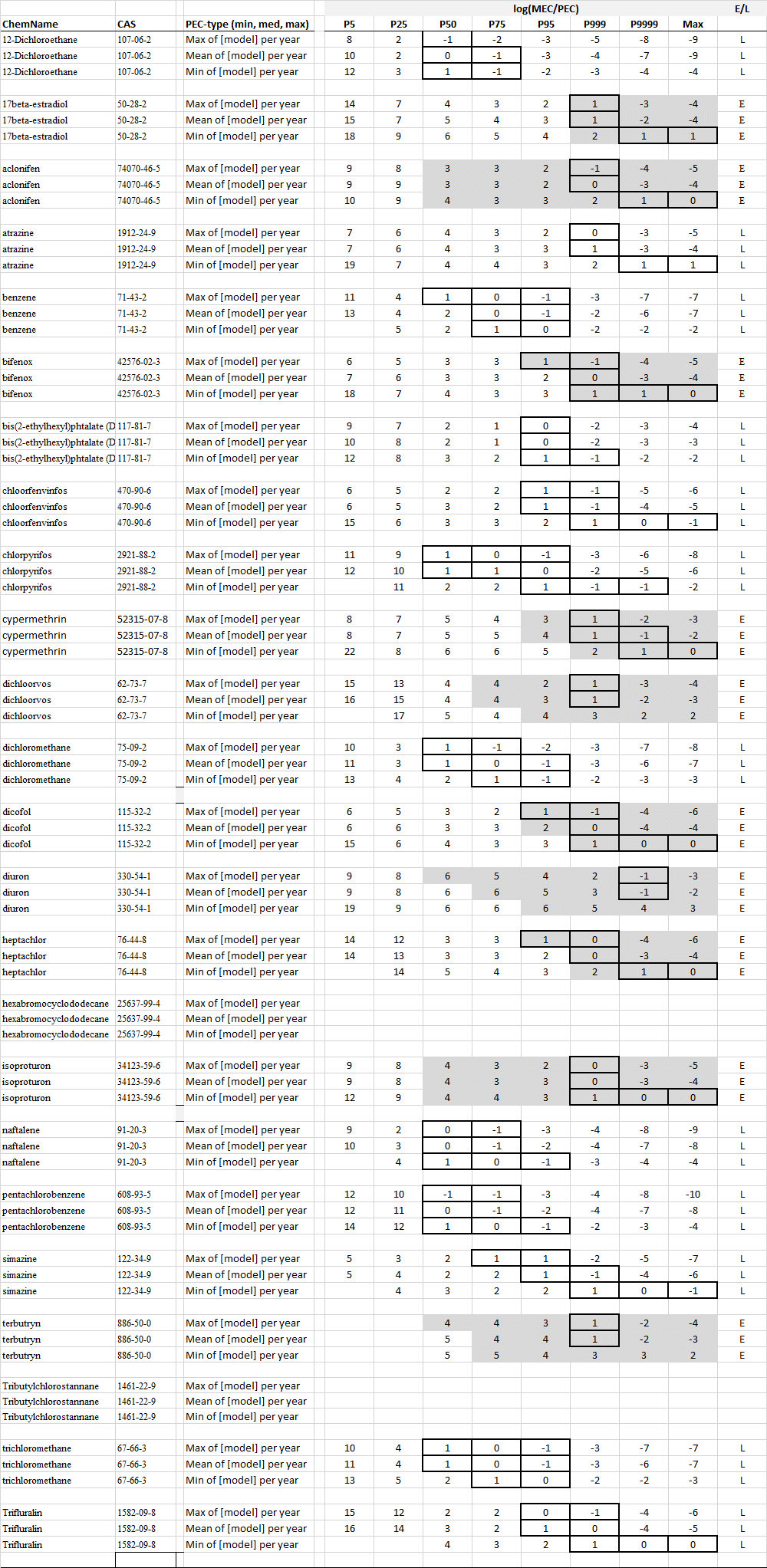


1. Alignment of ecological and chemical pollution data
2. **Aligning chemical and ecological data**

The WISE ecological status data, containing data for functional elementary catchments (FECs) were spatially aligned with the chemical pollution data for 35,406 E-HYPE hydrological units. As one E-HYPE units can relate to multiple FECs, the alignment of WISE and E-HYPE yielded 46,979 units with both PECs and ecological status data. The overlapping units are further referred to as sites or water bodies.

**2. Numbers of sites per ecological status class and broad river types**

The distributions of sites over ecological status classes and broad river types[^22^](#_ENREF_22) (RT), counted after aligning the chemical pollution data with the WISE data, is shown in Supplementary Table S6. The highest number of sites was present for good, moderate and poor ecological status, with relatively lower numbers for the high and bad ecological status classes. The numbers of sites per class, or broad river type + ecological status class, are considered sufficient for the present study.

Supplementary Table S6. Number of sites with aligned X (chemical pollution) and Y (ecological status) data, specified towards ecological status classes and broad river types. The boundary between the class “2_good” and “3_moderate” marks (1) a significant deviation from type-specific reference conditions and (2) the obligation to plan measures to reach (at least) the good status. River types are defined as follows: RT1=Very large rivers; RT2=Lowland brooks and streams with catchment size smaller than 100 km^2^; RT3=Lowland streams and rivers with catchment size 100-10,000 km^2^; RT4=Siliceous mid-altitude brooks and streams with catchment size smaller than 100 km^2^; RT5=Siliceous mid-altitude streams and rivers with catchment size 100-10,000 km^2^; RT6=Calcareous mid-altitude brooks and streams with catchment size smaller than 100 km^2^; RT7=Calcareous mid-altitude streams and rivers with catchment size 100-10,000 km^2^; RT8=Highland and glacial rivers; RT9=Mediterranean perennial; RT10=Mediterranean temporal or very small brooks.


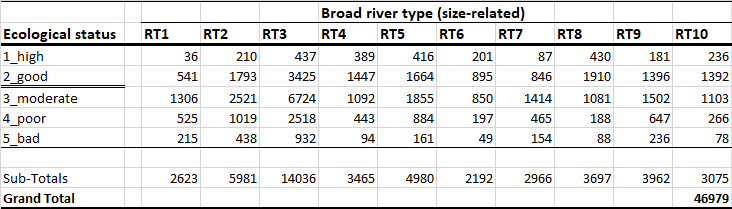


1. Patterns in the chemical pollution data
2. **Relationship between HI*_Median-EC50_* and msPAF*_EC50_***

The quantitative mixture pressure metrics used in the study, Steps 3 and 4 (HI*_Median-EC50_* and msPAF*_EC50_*), are derived from the same set of curated ecotoxicity test data[^7^](#_ENREF_7), but they are conceptually different. As explained in the main text, HI=Hazard Index, and msPAF=multi-substance Potentially Affected Fraction of species. The HI-approach is based on summation of risk quotients over all compounds, whereby it is implicitly assumed that concentration-impact relationships are linear. This is, however, not the case. Such relationships are sigmoidal in shape, they are referred to as species sensitivity distribution (SSD), and they are commonly described by log-normal (or similarly shaped) cumulative distribution functions of laboratory toxicity (sensitivity) data (such as the no-observed effect concentration [NOEC] or the 50%-effect concentration [EC50])[^23^](#_ENREF_23). We determined the relationship between both metrics, to evaluate whether and how the conceptual difference between HI and msPAF might matter for the quantitative assessments of the case study (Step 3 and 4).

A near-linear relationship between these metrics was found for HI ranges between approx. 0.01 and 10 (Supplementary Fig. S1). For the non-linear part of the association, the HI-values can span many orders of magnitude (X-axis), whilst the mixture toxic pressure – expressing the fraction of species affected – is conceptually and numerically limited to values ranging from 0 – 1 (between none and all species likely affected, Y-axis). As SSDs are commonly sigmoidal and are limited to the range of 0 to 100% of species potentially affected, the msPAF-approach provides the conceptually better parameter to characterize the chemical pollution pressure. Therefore, the mixture toxic pressure (msPAF) was added in the main study as Step 4 as the key metric for studying the relationship between chemical pollution and ecological status.


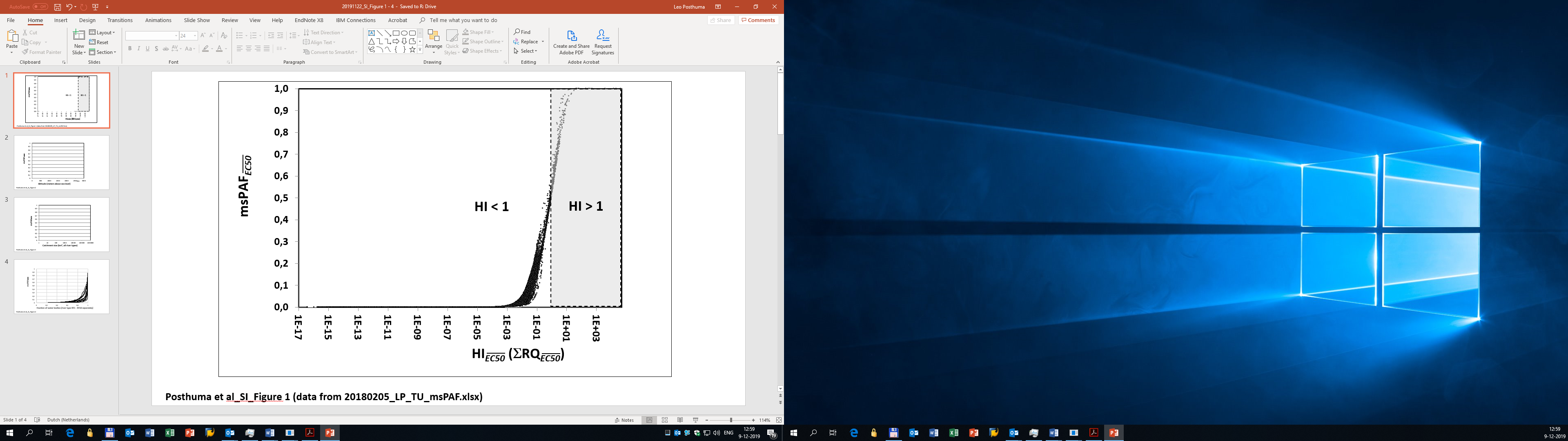


Supplementary Fig. S1. The relationship between the two mixture metrics. X = HI = hazard index, Y = msPAF (multi-substance Potentially Affected Fraction) = mixture toxic pressure (both based on EC50-data). The mixture toxic pressure equals 0.5 for HI=1, as follows from the definition of both metrics.

1. **Exploring some patterns of the mixture toxic pressure metric**

In addition to the evaluations of the predicted environmental concentrations vis-à-vis the measured concentrations of chemicals (see above), the set of mixture toxic pressure data was described in various ways to judge whether the observed patterns are similar to common-sense expectations (e.g., on expectations of emitted mass of chemicals at different sites in Europe, related to population density and economic activities).

1. Mixture toxic pressure and altitude. The relationship between altitude of the water body and the mixture toxic pressure metric (the year’s P95 of msPAF*_EC50_*) showed the lowest values at highest altitudes, with high mixture toxic pressure variability at moderate to low altitudes (Supplementary Fig. S2). This was expected from lower human population and associated, expected chemical emissions at higher altitudes.


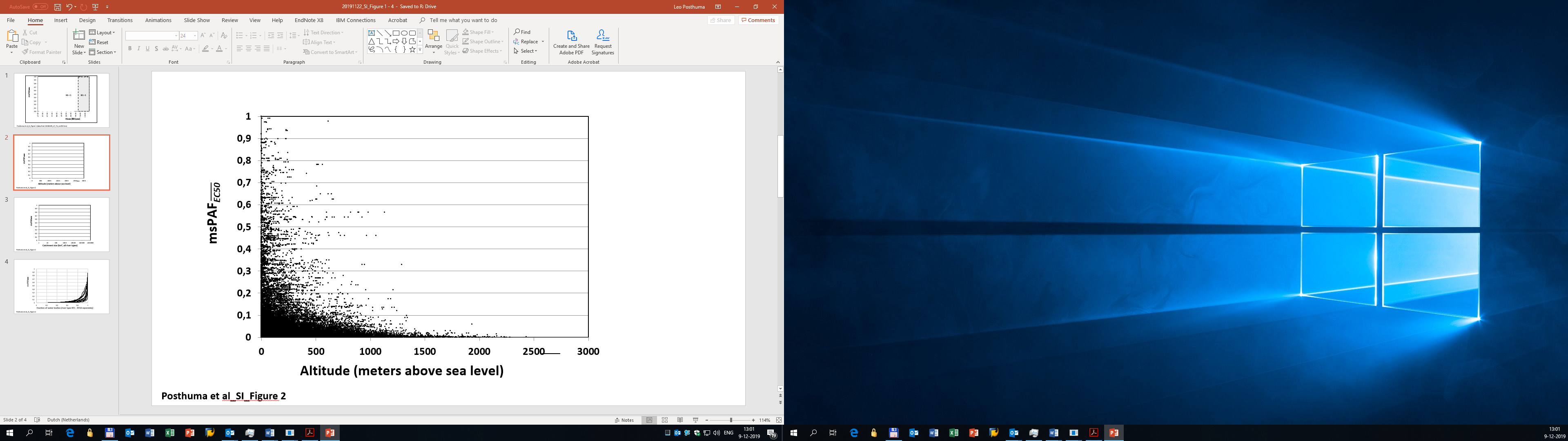


Supplementary Fig. S2. Geographical inspection of patterns in mixture toxic pressure: relationship to altitude.

2. Mixture toxic pressure and catchment size. The relationship between catchment size and the mixture toxic pressure metric showed the highest values for medium-sized catchments, and the lowest values for the smallest and largest sizes (Supplementary Fig. S3). The pattern is in line with the general expectation of lower pollution in cases of likely lower land-use- and population intensities (smallest catchments) and higher dilution (largest catchments).


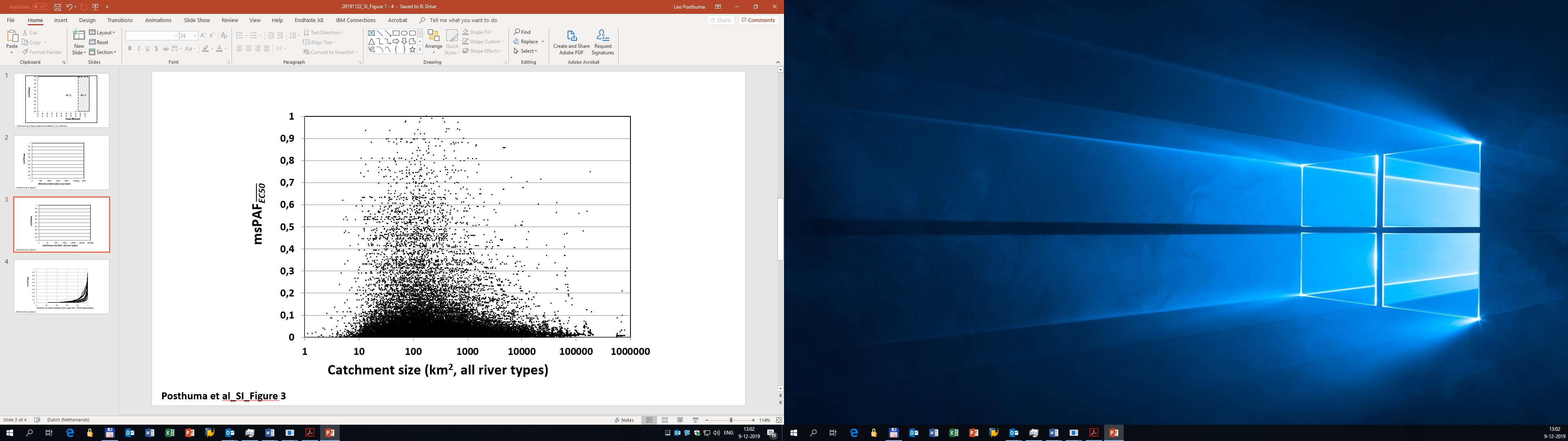


Supplementary Fig. 3. Geographical inspection of patterns in mixture toxic pressure: relationship to catchment size.

3. Mixture toxic pressure and broad river type. The distribution of mixture toxic pressure values for all broad river types is highly skewed, with relatively few sites exhibiting relatively high values (Supplementary Fig. S4). The broad river types slightly differ, but the cumulative distributions share the same shape without showing an exact overlay. This means, that some broad river types are – on average – potentially more affected than others by chemical pollution.


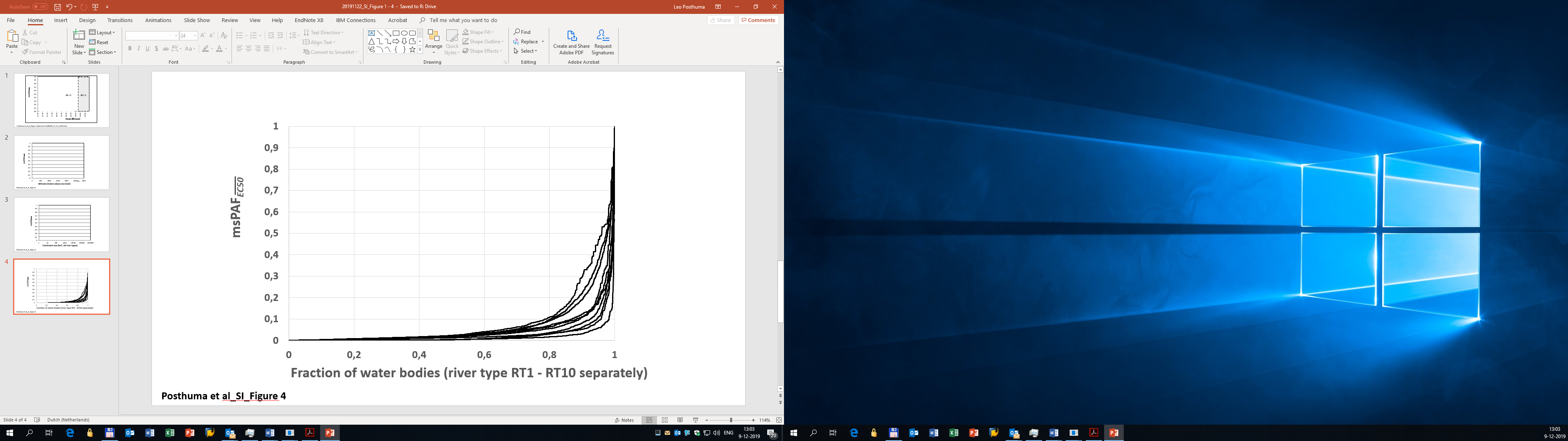


Supplementary Fig. S4. Cumulative distributions of the mixture toxic pressure metric for the distinct broad river types. The most polluted three river types (upper three distribution curves) are RT10 (Mediterranean temporal or very small rivers [brooks]), RT2 (lowland brooks and streams, catchment size <100 km^2^) and RT3 (lowland streams and rivers, catchment size 100-100,000 km^2^), followed by relatively similar distributions of chemical pollution in remaining river types, apart from RT8 (highland and glacial rivers) as relatively least polluted river type (lowest distribution curve).

1. Collinearity test of chemical pollution and other pressures

The final interpretation of the case study outcomes can be biased if statistical associations can also be caused by pressures covarying with the chemical pollution metric. Collinearity of mixture toxic pressure with other pressures might be present due to e.g. land uses that induce multiple pressures. Collinearity issues may be of limited importance for large-scale data sets in which all kinds of pressure combinations occur. We explored whether collinearity between chemical pollution and other pressures might affect the final interpretation of the case study outcomes.

Approach. Using the pairwise Spearman correlation coefficient, we tested mixture toxic pressure metric for collinearity with other stressor variables (urban and agricultural land use in the riparian zone[^24^](#_ENREF_24), human alteration of the mean annual river flow and the river base-flow index[^25^](#_ENREF_25), and dissolved inorganic nitrogen and total phosphorus concentration[^26^](#_ENREF_26)).

Results. Across all variables, the pairwise correlation amounted to r=0.11 on average including the data of all river types combined. Individual correlations based on type-specific analyses did not exceed r=0.40, with the exception of siliceous upland brooks and rivers showing up to r=0.57 with the stressors riparian land use and nutrient pollution.

Implication. The absence of considerable correlations between the mixture toxic pressure metric and the other pressures suggests that the statistical results of the final analyses made in the main paper (i.e., an increase in mixture toxic pressure limits maintaining or reaching high or good ecological status) is not biased by covariation between mixture toxic pressure and the other pressures that were evaluated.

1. Evaluation of other options to summarize PECs over the year

The assessments in Figures 1, 2 and 3 of the main manuscript were derived from the mean concentration of chemicals within a year (based on PEC-values), following the practices of the formal implementation of the WFD in assessing chemical pollution data (monitored chemical concentrations), but alternative choice to summarize exposure are possible. For example, the water quality can also be judged by assessing minimum or maximum concentrations, or the P95-PEC of a year. The latter two approaches imply that peak concentrations of e.g. pesticides are considered, and not ‘averaged out’ by determining yearly mean concentration. We investigated the outcomes of the assessments made in Figure 1, 2 and 3 of the main text by comparing the results obtained by using mean concentrations within a year with those of the minimum and the maximum concentration of a year (Supplementary Fig. S5), and summarized the outcome in a table (Supplementary Table S7).

Supplementary Fig. S5 shows that the percentage of water bodies for which the mixture HI*_EQS_* < 1 (representing water bodies for which the mixture exposure does not exceed the protection level represented by the environmental quality standards) decreases slightly when judging exposures with minimum, mean and maximum concentrations of a year, respectively. The percentages of water bodies sufficiently protected against impacts of mixtures of the 24 priority substances decreases from 32% to 26% and 25%. When based on mixture assessments at the 50%-effect level of the calculated median-sensitive species, the percentages of water bodies with HI*_Median-EC50_* < 1 are 95%, 85% and 78%, respectively (representing water bodies for which the mixture exposure does not exceed the 50%-effect level for the median-sensitive species). The numbers of compounds that contribute to HI*_EQS_* >1 are generally larger than for HI*_Median-EC50_* > 1, as expected from the higher probability of that the exposure exceeds the no-effect level rather than the 50%-effect level. The pattern shown in Supplementary Fig. S5 and Supplementary Table S7 suggest that the outcomes of the assessment of the relationship between mixture exposure and ecological status (main manuscript, Figure 4 and Table 1) may only be slightly affected by considering alternative choice for characterizing exposure: the percentage of sites affected at HI*_Median-EC50_* < 1 decreases marginally, from 85% to 78%, between using mean and maximum concentrations. The final conclusion, that chemical pollution affects ecological status, is not affected by a methodologically necessary choice on the approach chosen to summarize the exposure variability over a year (the summary of site PEC-data).


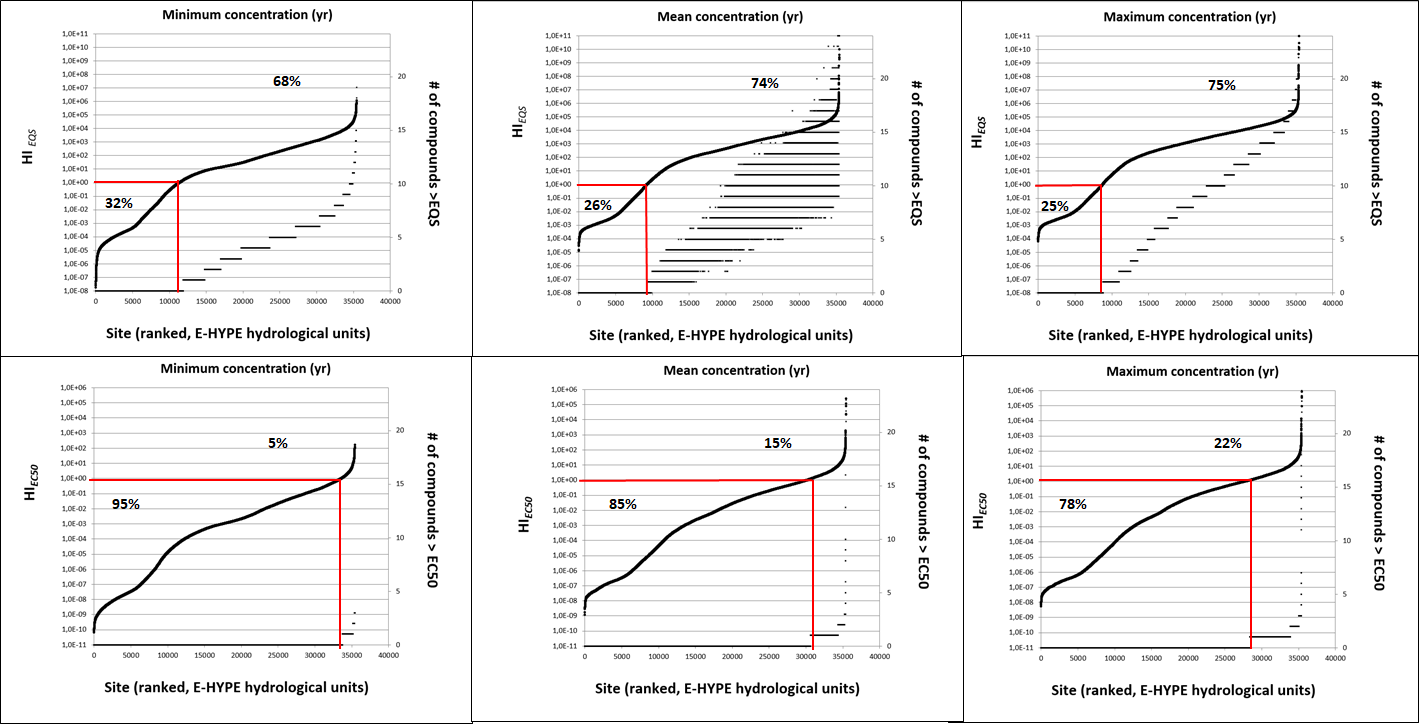


Supplementary Fig. S5. The assessments of main manuscript Figures 1, 2 and 3 based on minimum (left), mean (middle) and maximum (right) concentrations predicted within a year for all water bodies, and when judged vis-à-vis environmental quality standards (top) and across-species median-EC50s. The results of main manuscript Figure 1 are represented by the percentage boxes, representing the percentages of water bodies for which HI<1. The results of main manuscript Figures 2 and 3 are identical to the middle panels.

Supplementary Table S7. Percentage of water bodies for which the concentration of one compound exceeds its environmental quality standard (TU*_i_* > 1; TU=Toxic Unit of a chemical) and for which the mixture exposure exceeds the mixture exposure standard (HI > 1), for judgments based on exceedances of protective standards (EQS, top rows) and on exceedance of the EC50 for the median-sensitive species (Median-EC50), for the various ways to summarize exposure data (minimum, mean and maximum concentration in a year).

| **Evaluation** | **Concentration (in yr)** | **TU***_i_***>1** | **HI > 1** |
| --- | --- | --- | --- |
| EQS | Minimum | 58% | 68% |
|  | Mean | 67% | 74% |
|  | Maximum | 69% | 75% |
| Median-EC50 | Minimum | 1% | 5% |
|  | Mean | 3% | 15% |
|  | Maximum | 4% | 22% |

**REFERENCES**
